# Electronic State Spectroscopy of Nitromethane and Nitroethane

**DOI:** 10.1021/acs.jpca.2c08023

**Published:** 2023-02-02

**Authors:** Luiz V. S. Dalagnol, Márcio H. F. Bettega, Nykola C. Jones, Søren V. Hoffmann, Alessandra Souza Barbosa, Paulo Limão-Vieira

**Affiliations:** † Departamento de Física, Universidade Federal do Paraná, Caixa Postal 19044, 81531-980Curitiba, Paraná, Brazil; ‡ ISA, Department of Physics and Astronomy, Aarhus University, Ny Munkegade 120, DK-8000Aarhus C, Denmark; § Atomic and Molecular Collisions Laboratory, CEFITEC, Department of Physics, NOVA School of Science and Technology, Universidade NOVA de Lisboa, 2829-516Caparica, Portugal

## Abstract

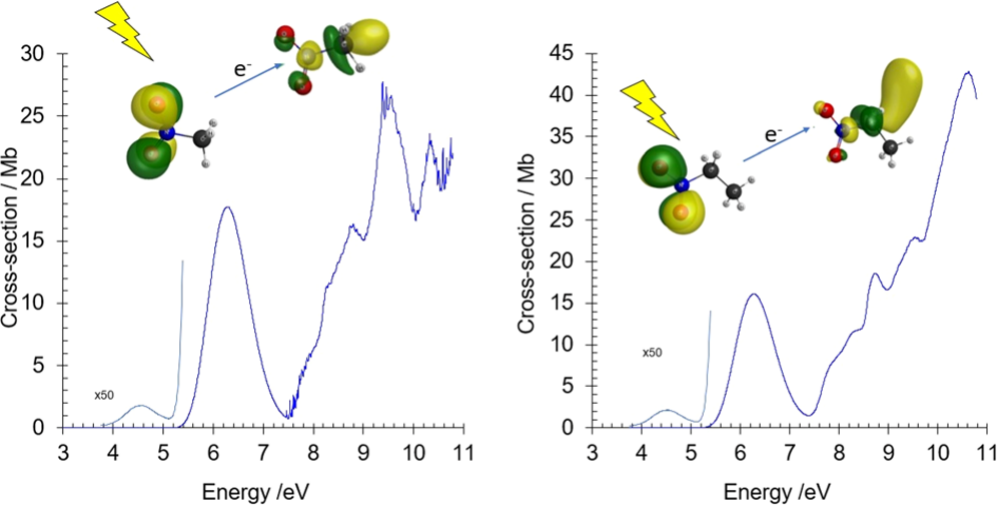

High-resolution photoabsorption cross-sections in the 3.7–10.8 eV energy range are reinvestigated for nitromethane (CH_3_NO_2_), while for nitroethane (C_2_H_5_NO_2_), they are reported for the first time. New absorption features are observed for both molecules which have been assigned to vibronic excitations of valence, Rydberg, and mixed valence-Rydberg characters. In comparison with nitromethane, nitroethane shows mainly broad absorption bands with diffuse structures, which can be interpreted as a result of the side-chain effect contributing to an increased number of internal degrees of freedom. New theoretical quantum chemical calculations performed at the time-dependent density functional theory (TD-DFT) level were used to qualitatively help interpret the recorded photoabsorption spectra. From the photoabsorption cross-sections, photolysis lifetimes in the terrestrial atmosphere have been obtained for both compounds. Relevant internal conversion from Rydberg to valence character is noted for both molecules, while the nuclear dynamics of CH_3_NO_2_ and C_2_H_5_NO_2_ along the C–N reaction coordinate have been evaluated through potential energy curves at the TD-DFT level of theory, showing that the pre-dissociative character is more prevalent in nitromethane than in nitroethane.

## I Introduction

Nitromethane (CH_3_NO_2_) is a simple organic-nitro compound used as a solvent in the pharmaceutical and chemical industries, whereas from the research point of view, it can be regarded as a prototypical molecule to be used as a benchmark system for high-level computational modeling of molecular energetics and structural properties.^1^ It can be expected to act as a human carcinogenic agent and is therefore of biological relevance, and it may play a role in the chemistry of the Earth’s troposphere and stratosphere following the reaction of CH_3_ radicals with NO_*x*_ and other oxides of nitrogen,^1^ e.g., nitrous oxide and N_2_O. Also, CH_3_NO_2_ belongs to the class of molecules with typical characteristics of explosives, propellants, and even has military use. Thus, clarification of the complex processes involved in its detonation and propellant behavior requires detailed knowledge of the electronic state spectroscopy of both neutral and ionic species.^2^ Spectroscopic studies involving CH_3_NO_2_ have been reported on several occasions in the past and include the experimental methods: vacuum ultraviolet (VUV) absorption,^1,3^ electron impact excitation,^4−6^ Raman and infrared absorptions,^3,7−10^ He(I) and He(II) photoelectron spectroscopies,^11−14^ photodissociation spectroscopy,^15,16^ and rate constants for its reactions with hydroxyl radicals.^17,18^ We also note theoretical studies on the structure and electronic properties of nitromethane^19^ and the elastic scattering of low-energy electrons.^20^ Antunes et al.^21^ and Alizadeh et al.^22^ have reported negative ion formation in electron transfer and dissociative electron attachment experiments to nitromethane, respectively, where high-energy resonances (>4 eV) of several fragment anions have been assigned to electronically excited temporary negative ion states (including Rydberg excitation). Such core-excited resonances can be compared to the parent Rydberg states in the VUV spectrum.

Nitroethane (C_2_H_5_NO_2_) is a relevant industrial chemical compound used in the production of nitroalcohols, pharmaceuticals, and organic synthesis, as well as a solvent for celluloses, resins, and waxes.^23^ It can be degraded by reactions with ^•^OH radicals in the atmosphere yielding an estimated half-life for such reactions in air of a little more than 100 days. Relevant to the present work, there are a few studies involving the absorption spectrum in the 3.4–5.6 eV photon energy region,^24^ Raman^10^ and infrared^8^ spectroscopies, He(I) and He(II) photoelectron spectroscopies,^14^ and the rate constants for nitroethane reactions with hydroxyl radicals.^18^

In Section II, we present a brief description of the experimental methodology, and in Section III, the computational details of the calculations used to help the interpretation of experimental data are shown. In Section IV, a brief summary of the structures of CH_3_NO_2_ and C_2_H_5_NO_2_ is given, and Sections V–VII include a comprehensive description of the electronic state spectroscopy of nitromethane and nitroethane in the 3.7–10.8 eV photon energy region and compares the present data with other absolute photoabsorption cross-sections where possible. Potential energy curves along the C–N coordinate are discussed in Section VIII. Absolute photoabsorption cross-sections are compared with previous work, and the data used to calculate the photolysis rates of CH_3_NO_2_ and C_2_H_5_NO_2_ from in the Earth’s atmosphere are presented in Section IX. We finish with Section X by including a brief summary of our results and conclusions.

## II Experimental Method

The apparatus used to record high-resolution VUV photoabsorption spectra of nitromethane and nitroethane has been described before^25^ and is based on that described by Eden et al.^26^ Briefly, it consists of a static absorption gas cell and a photon multiplier tube (PMT) for recording the transmitted light through the cell. The experimental data were obtained at the AU-UV beamline at ASTRID2 storage ring facility, Aarhus University, Denmark.

The absolute photoabsorption cross-sections, in units of megabarn (1 Mb ≡ 10^–18^ cm^2^), were measured with monochromatized synchrotron radiation in the wavelength region 115–330 nm (3.7–10.8 eV). This yielded an effective energy resolution of 3 meV (full width at half-maximum (FWHM)) at the mid-point of the energy range studied. The absorption gas cell is filled with vapor of the molecular compound under investigation, and the transmission windows (MgF_2_) used to enclose the cell set the lower-wavelength limit of detection (115 nm). The absolute pressure in the target cell was measured with a capacitance manometer (Chell CDG100D) and to avoid any saturation effects in the data recorded, the absorption cross-sections were carefully measured using a pressure within the range 0.08–1.22 mbar, which was appropriate for the local cross-section, to have attenuations of 50% or less. Cross-sections are measured through attenuation of the incident photon beam and background scans with the cell evacuated, and evaluated according to the Beer–Lambert law: *I*_t_*= I*_0_ exp(−σ*Nl*), where *I*_t_ and *I*_o_ are the light intensities transmitted through the gas sample with and without the sample, respectively, *σ* is the absorption cross-section, *N* is the target number density, and *l* is the absorption path length (15.5 cm). The synchrotron beam ring current was monitored throughout the collection of each spectrum and compensation for the beam decay in ASTRID 2 is achieved by running in a “top-up” mode allowing the light intensity to be kept quasi-constant. The small variations (ca. 2–3%) of the incident flux are normalized to the beam current in the storage ring. This methodology allows us to determine the accuracy of the photoabsorption cross-sections to within ±5%. Accurate cross-section values are obtained by recording the VUV spectrum in small (5 or 10 nm) sections, allowing an overlap of at least 10 points between the contiguous sections. The proposed assignments of the recorded absorption spectral features of nitromethane and nitroethane are listed in Tables 2, 3, 5, and 6, and Tables 5 and 7, respectively.

The liquid samples of nitromethane and nitroethane used in the VUV photoabsorption measurements were purchased from Sigma-Aldrich, with a stated purity of ≥99 and 99.5%. The samples were degassed through repeated freeze–pump–thaw cycles.

## III Theoretical Method

The optimized geometries of nitromethane and nitroethane neutral and ionic electronic ground-states (Supporting Information (SI) Figures S1 and S2, and S3 and S4) have been calculated at the DFT level of theory, where the B3LYP functional and Dunning’s augmented correlation consistent valence double-ζ (aug-cc-pVDZ) basis set, as implemented in the package GAMESS-US,^27^ were used. The excited electronic states were obtained for the ground-state optimized molecular geometries of both molecules, employing TD-DFT^28,29^ with a B3LYP functional and the aug-cc-pVDZ basis set.

At room temperature, nitromethane and nitroethane have two conformers, *eclipsed* and *staggered*, both being of *C*_S_-symmetry in their electronic ground-states. The difference between conformers is due to the torsion of the CH_3_ group, where for the *eclipsed* conformer the symmetry plane contains the NO_2_ group while for *staggered* the plane is normal to NO_2_ and bisecting the ONO angle (Figures S1 and S2). As far as nitromethane is concerned, there is a general consensus that the relative energy between conformers is very small and this is due to the barrier of internal rotation of the methyl group, which has been reported to be 0.26 meV (6 kcal mol^–1^).^30,31^ A literature survey reveals the majority of earlier works report the *staggered* conformer to be lower in energy,^32−34^ albeit a few others claiming that it is the *eclipsed* conformer.^35,36^ Nonetheless, our calculations using DFT/B3LYP/aug-cc-pVDZ show the *eclipsed* conformer to be marginally lower in energy, with that difference among conformers to be ∼0.1 × 10^–4^ eV, whereas for nitroethane, that difference is 4 meV. Thus, at room temperature, equal populations of both conformers for each molecule are considered.

**Figure 1:**
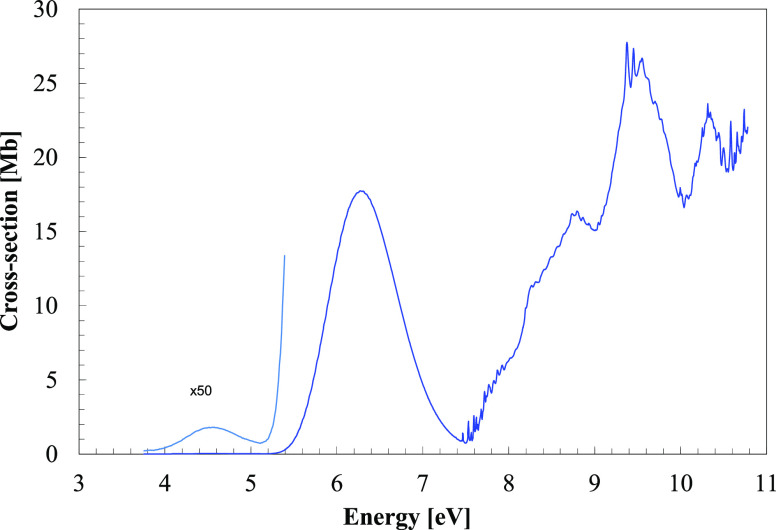
VUV photoabsorption spectrum of CH_3_NO_2_ in the 3.7–10.8 eV energy region.

A complete list of calculated vertical excitation energies for both neutral conformers of CH_3_NO_2_ and C_2_H_5_NO_2_ molecules is presented in Tables S1 and S2, respectively, while in the main body of text, we show only the dominant vertical excitation energies for the *eclipsed* conformers. Note that the calculated vertical excitation energies of both conformers (Tables S1 and S2), and the character of the dominant electronic transitions in the absorption spectra (Figures 1 and 4) are very similar for both conformers of each molecule. Representation of a selection of molecular orbitals of nitromethane and nitroethane are shown in Figures S5 and S6.

## IV Structure and Orbital Properties of CH_3_NO_2_ and C_2_H_5_NO_2_

### IV.A Nitromethane, CH_3_NO_2_

Nitromethane (CH_3_NO_2_) has *C*_S_ symmetry in its ground electronic state, and the calculated outermost valence electronic configuration of the *X̃*^1^*A*^′^ state is: ··· (9a′)^2^ (10a′)^2^ (11a′)^2^ (2a″)^2^ (3a″)^2^ (12a′)^2^ (13a′)^2^. The character of the ground-state MOs (see Figure S5) shows that the highest occupied molecular orbital (HOMO), 13a′, and the (HOMO-1), 12a′, are O 2p lone pair orbital (*n̅*_*O*_) in the molecular plane, the latter also showing σ_CN_ bonding character. The third and the fourth highest occupied molecular orbitals (HOMO-2), 3a″, and (HOMO-3), 2a″, are π_N=O_ in character.

**Figure 2:**
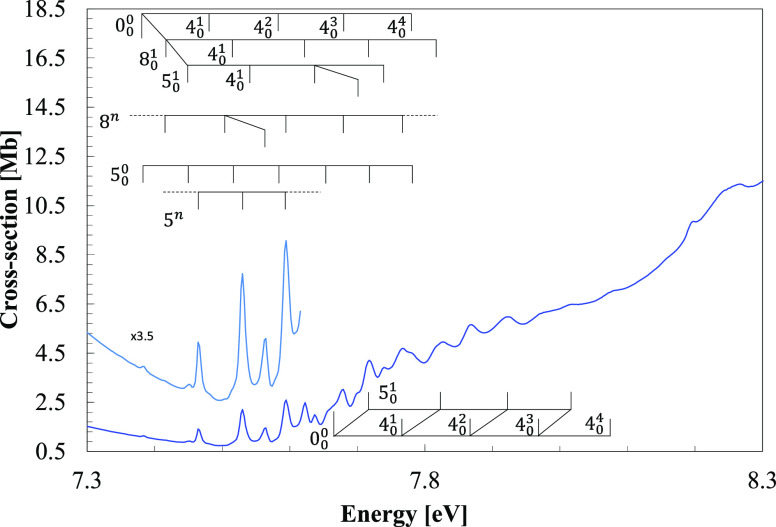
VUV photoabsorption spectrum of CH_3_NO_2_ in the 7.3–8.3 eV energy region with labeled vibrational series.

The photoabsorption features (Figures 1 and 2) have been mainly assigned to electronic excitations, due to the promotion of an electron from these MOs to unoccupied valence, Rydberg, and mixed valence-Rydberg characters (see Table 1 for the calculated dominant excitation energies and oscillator strengths). The fine structure in the photoabsorption spectrum has been assigned to vibronic transitions (Tables 2 and 3) from the main fundamental vibrational modes available from Raman and infrared spectroscopies data.^7,8,10^ Assignments of these modes from the energies in the electronic ground-state are 0.171 eV for NO_2_ symmetric stretching, *v*_3_^′^(*a*^′^), 0.114 eV for C–N stretching, *v*_4_^′^(*a*^′^), 0.081 eV for NO_2_ symmetric bending, *v*_5_^′^(*a*^′^) and 0.059 eV for NO_2_ rocking, *v*_8_^′^(*a*^′^). For the Rydberg character of the electronic transitions, the vibrational modes have been assigned according to the experimental information from He(I) photoelectron spectroscopy of the NO_2_ symmetric bending mode to 0.065 eV (1 ^2^*A*_1_) and 0.068 eV (1 ^2^*A*_2_) from the work of Rabalais,^12^ and 0.059 eV (1 ^2^A′) and 0.067 eV (1 ^2^A″) from Mok et al.^14^ Additionally, vibrational frequencies of CH_3_NO_2_ neutral and ionic electronic ground-states have been calculated at the B3LYP/aug-cc-pVDZ level of theory (Table S3).

**Table 1:** Calculated Dominant Vertical Excitation Energies (TD-DFT/B3LYP/aug-cc-pVDZ) and Oscillator Strengths of CH_3_NO_2_, Compared Where Possible with the Corresponding Experimental Data and Other Work in the Literature (Energies in eV)^a^

nitromethane (CH_3_NO_2_)							
state	*E* (eV)	*f*_L_	dominant excitations	*E*_exp._ (eV)^b^	cross-section (Mb)	*E* (eV)^1^	*E* (eV)^3^
X̃ ^1^A′							
2 ^1^A″	4.605	0.00002	π^*^(4*a*^″^) *← n̅*_*O*_*/*σ_CN_(12*a*′) (100%)	4.550	0.04	4.5	
2 ^1^A′	6.879	0.15459	π^*^(4*a*^″^) *←* π(3*a*^*″*^) (90%)	6.271	17.74	6.25	6.27
3 ^1^A′	7.100	0.00037	3*s*(14*a*^′^) *← n̅*_*O*_(13*a*^′^) (95%)	7.637	2.59	7.44	7.531
4 ^1^A′	7.510	0.03627	3*s*(14*a*^′^) *← n̅*_*O*_/σ_CN_(12*a*^′^) (97%)	8.38(3)(s)	12.13	8.07	8.257
6 ^1^A′	8.223	0.02625	3*p*_*x*_*/*σ(16*a*^′^) ← *n̅*_*O*_(13*a*^′^) (72%) + *3p*_*y*_(15*a*^′^) *← n̅*_*O*_(13*a*^′^) (22%)	7.92(5)	5.98	7.8	7.974
9 ^1^A′	8.851	0.10584	π^*^(4*a*^*″*^) ← π_NO_(2*a*^*″*^) (70%) + 3*p*_*x*_/σ(16*a*^′^) *← n̅*_*O*_/σ_CN_(12*a*^′^) (21%)	8.803	16.33	8.3	8.531
12 ^1^A′	9.702	0.20343	σ_CN_^*^(17*a*^′^) *← n̅*_*O*_/σ_CN_(12*a*^′^) (79%) + 4*s/*4*p*_*y*_(19*a*^′^) *← n̅*_*O*_(13*a*^′^) (14%)	9.450	27.35		9.368
19 ^1^A′	10.696	0.03473	*4s/*5*p*_*z*_(20*a*^′^) *← n̅*_*O*_/σ_CN_(12*a*^′^) (93%)	10.315	23.63		10.347
18 ^1^A″	10.960	0.02397	4*s*/π^*^(20*a*^′^) *← n̅*_*O*_/σ_CN_(12*a*^′^) (90%)	10.739	22.32		10.798

a See text for details.

b The last decimal of the energy value is given in parentheses for these less-resolved features.

**Table 2:** Energy Positions (in eV) of Progressions and Vibrational Analysis of Features Observed in the Photon Energy Range 7.3–8.3 eV of CH_3_NO_2_

energy^a^	assignment	Δ*E* (υ_3_′)	Δ*E* (υ_4_′)	Δ*E* (υ_5_′)	Δ*E* (υ_8_′)	ref (3)
3*s*(14*a*′) ← *n̅*_*O*_/σ_CN_(12*a*^′^)						
7.382	0_0_^0^					
7.41(5)(s,w)	8^*n*^					
7.420	8_0_^1^				0.038	
7.453	5_0_^1^			0.071		
7.464	5^*n*^					
7.48(3)(s)	4_0_^1^		0.103			
7.50(1)(s,w)	5_0_^1^ + 8_0_^1^/8^*n*+1^		0.086		0.048	
7.51(2)(s)	8_0_^1^ + 4_0_^1^		0.092			
7.51(9)(s)	5_0_^2^/3_0_^1^	0.139		0.066		
7.530	5^*n*+1^			0.066		7.531
7.53(9)(s)	5_0_^1^ + 4_0_^1^		0.086			
7.565	5_0_^2^ + 8_0_^1^/3_0_^1^ + 8_0_^1^				0.046	7.563
7.57(9)(s)	4_0_^2^		0.096			
7.58(6)(s)	5_0_^3^			0.067		
7.595	5_0_^1^ + 4_0_^1^ + 8_0_^1^/8^*n*+2^/5^*n*+2^		0.094	0.065	0.056	7.594
7.623	8_0_^1^ + 4_0_^2^		0.111		0.044	7.622
7.637	5_0_^1^ + 4_0_^2^/3*s*(14*a*′) ← *n̅*_*O*_(12*a*^′^)		0.098			
7.65(6)(s)	5_0_^4^/3_0_^2^	0.137		0.070		7.656
7.679	4_0_^3^/5_0_^1^ + 4_0_^2^ + 8_0_^1^/8^*n*+3^		0.100/0.084		0.042	7.675
7.69(9)(s)	5_0_^2^ + 4_0_^2^			0.062		7.692
7.718	5_0_^5^		0.095	0.062		7.715
7.73(7)(s)	5_0_^1^ + 4_0_^3^		0.100			7.742
7.766	5_0_^1^ + 4_0_^3^ + 8_0_^1^/8^*n*+4^		0.087		0.029	7.769
7.77(6)(s)	4_0_^4^		0.097			
7.82(0)(s)	8_0_^1^ + 4_0_^3^		0.102			7.820
7.78(3)(s)	5_0_^6^/3_0_^3^	0.127		0.065		
		0.134	0.096	0.066	0.043	
3*p*_*y*_(15*a*′) *← n̅*_*O*_(13*a*^′^) + 3*p*_*x*_*/*σ_CN_ (16*a*′) *← n̅*_*O*_(13*a*′)						
7.66(3)(s)	0_0_^0^					
7.766	4_0_^1^		0.103			7.769
7.718	5_0_^1^			0.055		
7.82(5)(b)	5_0_^1^ + 4_0_^1^		0.107			7.820
7.870	4_0_^2^		0.104			7.869
7.92(5)(b)	5_0_^1^ + 4_0_^2^		0.100			7.918
7.97(3)(s,w)	4_0_^3^		0.103			7.974
8.01(7)(s,w)	5_0_^1^ + 4_0_^3^		0.092			8.023
8.07(7)(s,w)	4_0_^4^		0.104			8.078
			0.102	0.055		

a (s) shoulder structure; (w) weak feature; (b) broad structure (the last decimal of the energy value is given in parentheses for these less-resolved features).

**Table 3:** Proposed Vibrational Assignments of CH_3_NO_2_ Valence and Rydberg Series Converging to the Ionic Electronic Ground (13a′)^−1^ and First (12a′)^−1^ Excited States in the Photon Energy Range 9.0–10.8 (Energies in eV)^a^

energy^b^	assignment	Δ*E* (υ_3_′)	Δ*E* (υ_4_′)	Δ*E* (υ_5_′)	Δ*E* (υ_8_′)	ref (3)
*3p*_*x*_/σ_CN_(16*a*^′^) ← *n̅*_*O*_/σ_CN_(12*a*^′^) + π^*^ (4*a″*) *←* π(2*a*^*″*^)						
8.211	0_0_^0^					
8.268	8_0_^1^				0.057	8.257
8.313	4_0_^1^		0.102			8.302
8.38(3)(s)	4_0_^1^ + 5_0_^1^/3*s*(12*a*′)^−1^			0.070		8.345
			0.102	0.070	0.057	
8.420	3*p*(13*a*′)^−1^					8.437
8.492	5_0_^1^			0.072		8.478
8.565	5_0_^2^			0.073		8.568
8.637	5_0_^3^			0.072		8.687
8.70(4)(s,w)	5_0_^4^			0.067		
8.728	5_0_^2^ + 3_0_^1^	0.163				8.732
8.803	5_0_^3^ + 3_0_^1^/3*p*′(13*a*′)^−1^	0.166				8.792
8.869	5_0_^4^ + 3_0_^1^	0.165				8.862
8.930	5_0_^4^ + 3_0_^1^ + 5_0_^1^			0.061		
9.043	5_0_^4^ + 3_0_^2^	0.174				
9.08(3)(s)	3*p*(12*a*′)^−1^					
9.16(7)(s)	5_0_^1^			0.084		9.126
9.23(2)(s,w)	5_0_^2^			0.065		
9.32(2)(s,w)	5_0_^3^			0.090		
9.379	3*d*(13*a*′)^−1^					9.518
9.450	5_0_^1^/4p′(12*a*′)^−1^			0.071		
9.530	5_0_^2^			0.080		
9.552	4*s*(13*a*′)^−1^					9.541
9.62(6)(s)	5_0_^1^			0.074		
9.701	5_0_^2^			0.075		
9.77(0)(s)	4*p*(13*a*′)^−1^					9.761
9.85(2)(s,w)	5_0_^1^			0.082		
9.91(1)(s)	5_0_^2^/4*p*′(13*a*′)^−1^			0.059		9.806
9.995	5_0_^3^			0.084		
10.06(8)(b)	5_0_^4^			0.073		
10.445	4*p*(12*a*′)^−1^					10.439
10.629	3_0_^1^/6*p*′(13*a*′)^−1^	0.184				10.628
10.583	6*p*(13*a*′)^−1^/*6p*′(12*a*′)^−1^					10.573
10.656	5_0_^1^			0.073		
10.66(5)(s)	6*d*(13*a*′)^−1^					
10.763	4_0_^1^		0.098			
		0.170	0.098	0.074		

a See text for details.

b (s) shoulder structure; (w) weak feature; (b) broad structure (the last decimal of the energy value is given in parentheses for these less-resolved features).

The two lowest experimental adiabatic ionization energies, needed to calculate the quantum defects associated with transitions to Rydberg orbitals, are taken from the work of Rabalais^12^ to be 11.07 eV (13*a*′)^−1^ and 11.73 eV (12*a*′)^−1^.

### IV.B Nitroethane, C_2_H_5_NO_2_

Nitroethane (C_2_H_5_NO_2_) has *C*_S_ symmetry in its ground electronic state, and the calculated outermost valence electronic configuration of the *X̃*^1^*A*^′^ state is: ··· (2a″)^2^ (13a′)^2^ (14a′)^2^ (3a″)^2^ (4a″)^2^ (15a′)^2^ (16a′)^2^. The ground-state MOs’ character (Figure S6) shows that the highest occupied molecular orbital (HOMO), 16a′, is mainly the O 2p lone pair orbital (*n̅*_*O*_) in the molecular plane, with some σ_CC_ character, while the (HOMO-1), 15a′, is (*n̅*_*O*_) with σ_CN_ character. The third highest occupied molecular orbital (HOMO-2), 4a″, is π_N=O_ and (HOMO-3), 4a″, the O 2p lone pair orbital (*n*_*O*_) out of the molecular plane with σ_*C-H*_ character. Other molecular orbitals from which promotion of electrons have been assigned are (HOMO-4), 14a′, and (HOMO-5), 2a″, with (*n̅*_*O*_) and σ_CCN_, and π_N=O_ characters, respectively.

The photoabsorption features (Figures 4 and 5) have been assigned to electronic excitations from mainly these MOs to valence, Rydberg, and mixed valence-Rydberg character orbitals (Table 3). The information on the vibrational modes’ energies provided by infrared and Raman spectroscopies,^8,10^ was used to assign the excitations discernible in the nitroethane photoabsorption spectrum. Therefore, the available energies in the electronic ground-state are: 0.109 eV for C–N stretching, *v*_4_^′^(*a*^′^) and 0.077 eV for NO_2_ symmetric bending, *v*_5_^′^(*a*^′^), modes. Moreover, the vibrational mode assigned for the Rydberg character of the electronic transitions has been made on the experimental He(I) photoelectron data available from Mok et al.^14^ of the NO_2_ symmetric bending mode 0.063 eV (1 ^2^A″). Vibrational frequencies of C_2_H_5_NO_2_ neutral and ionic electronic ground-states were calculated at the B3LYP/aug-cc-pVDZ level of theory (see Table S3).

The two lowest experimental vertical ionization energies, needed to calculate the quantum defects associated with transitions to Rydberg orbitals, are taken from the work of Mok et al.^14^ to be 11.08 eV (16*a*′)^−1^ and 11.51 eV (15*a*′)^−1^.

## V Results and Discussion

**Figure 3:**
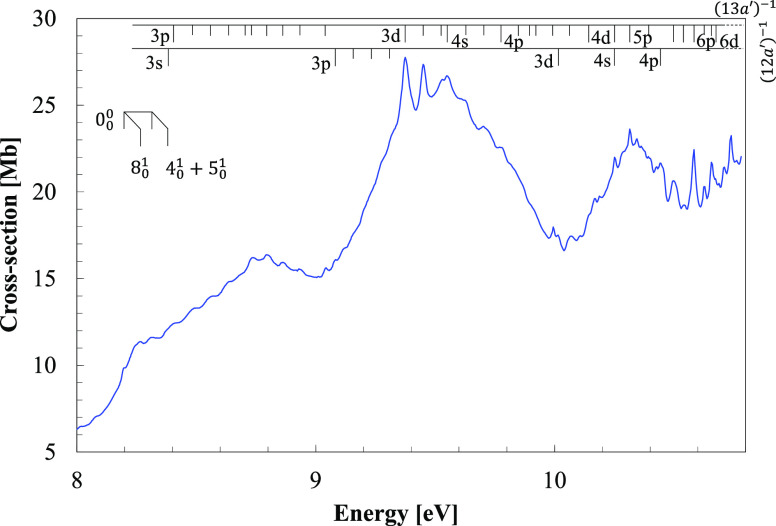
VUV photoabsorption spectrum of CH_3_NO_2_ in the 8.0–10.8 eV energy region with labeled Rydberg series converging to the ionic electronic ground and the first ionic electronic excited states.

**Figure 4:**
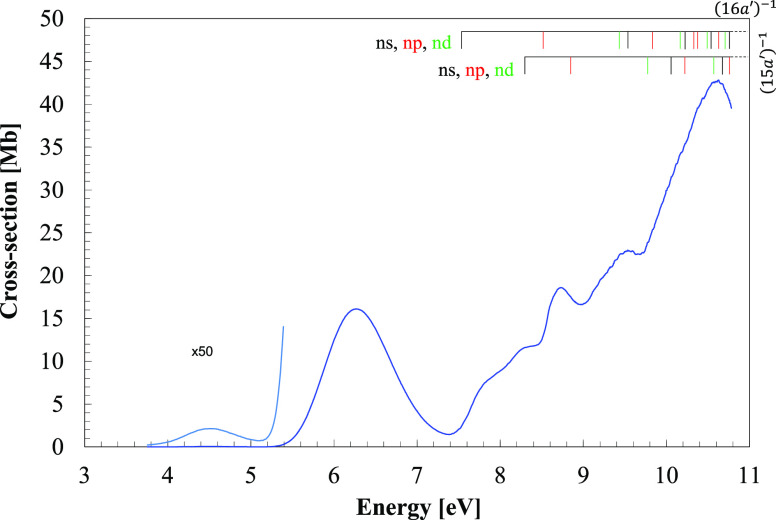
VUV photoabsorption spectrum of C_2_H_5_NO_2_ in the 3.7–10.8 eV energy region with labeled Rydberg series converging to the ionic electronic ground and the first ionic electronic excited states.

**Figure 5:**
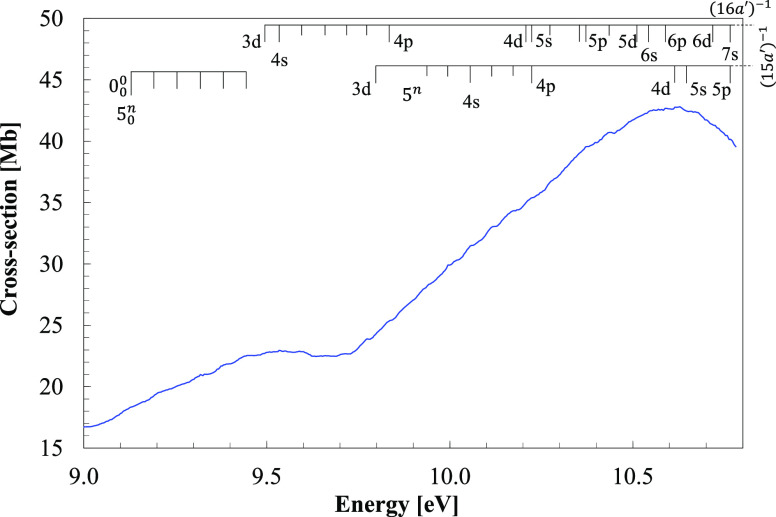
VUV photoabsorption spectrum of C_2_H_5_NO_2_ in the 9.0–10.8 eV energy region with labeled vibrational and members of Rydberg series.

The room temperature high-resolution VUV photoabsorption spectra of CH_3_NO_2_ and C_2_H_5_NO_2_ are shown in Figures 1 and 4, in the photon energy range 3.7–10.8 eV, while expanded views of the measured cross-sections are shown in Figures 2 and 3 for the former and Figure 5 for the latter molecular compound. The absorption bands are due to excitations from the *X̃*^1^*A*^′^ ground-state to valence, Rydberg, and mixed valence-Rydberg states (see Section VI) converging to the lowest-lying ionic states. Tables 1 and 4 show TD-DFT results for CH_3_NO_2_ and C_2_H_5_NO_2_ (vertical excitation energies and oscillator strengths) with the experimental results, where a good level of accord is noted. The spectra exhibit fine structures, which are much less pronounced in nitroethane given its higher number of internal degrees of freedom relative to nitromethane. This may then contribute to broadening and weakening of the vibronic absorption features. It is interesting to note that a similar effect has been observed in different fatty acids as the side chain is increased from propionic, to butyric and to valeric acids.^37^ Notwithstanding, these features have been assigned in nitromethane to NO_2_ symmetric stretching, *v*_3_^′^(*a*^′^), C–N stretching, *v*_4_^′^(*a*^′^), NO_2_ symmetric bending, *v*_5_^′^(*a*^′^), and NO_2_ rocking, *v*_8_^′^(*a*^′^), modes, mostly dominant above 7.3 eV, whereas in nitroethane to C–N stretching, *v*_4_^′^(*a*^′^) and NO_2_ symmetric bending, *v*_5_^′^(*a*^′^), modes above 9.1 eV. The photoabsorption features above 8 eV are mostly due to the overlap of different members of the Rydberg series converging to the ionic electronic ground and the first ionic electronic excited states. These, together with vibrational fine structure superimposed on them, make the spectra quite congested, and so the assignments are only partially labeled in Figures 3 and 5, only their energies indicated by vertical bars. The assignments are listed in Tables 2, 3, and 5.

**Table 4:** Calculated Dominant Vertical Excitation Energies (TD-DFT/B3LYP/aug-cc-pVDZ) and Oscillator Strengths of C_2_H_5_NO_2_, Compared Where Possible with the Corresponding Experimental Data and Other Work in the Literature (Energies in eV)^a^

nitroethane (C_2_H_5_NO_2_)					
state	*E* (eV)	*f*_*L*_	dominant excitations	*E*_exp._ (eV)^b^	cross-section (Mb)
X̃ ^1^A′					
2 ^1^A″	4.605	0.00006	π^*^(5*a*^*″*^) *← n̅*_*O*_/σ_CN_(15*a*^′^) (99%)	4.550	0.04
2 ^1^A′	6.789	0.11079	π^*^(5*a*^*″*^) *←* π(4*a*^*″*^) (86%)	6.256	16.10
5 ^1^A′	7.480	0.06883	π^*^(5*a*^*″*^) *←* n_O_/σ_CH_(3*a*^*″*^) (70%) + 3*s*(17*a*^′^) *← n̅*_*O*_/σ_CN_(15*a*^′^) (20%)	7.847	7.76
7 ^1^A′	8.087	0.02867	3*s*(19*a*^′^) *← n̅*_*O*_(16*a*^′^) (74%) + 3*s*(18*a*^′^) ← *n̅*_*O*_/σ_CN_(15*a*^′^) (20%)	8.271	11.48
12 ^1^A′	9.129	0.01501	4*s/*3*p*_*y*_*/3p*_*z*_(20*a*^′^) ← *n̅*_*O*_ (16*a*^′^) (81%)	8.725	18.60
17 ^1^A′	9.664	0.05147	π*(5*a″*) *←* π(2*a*^*″*^) (29%) + 3*s*(17*a*^′^) ← *n̅*_*O*_*/*σ_CC_(14*a*^′^) (49%)	9.44(6)	22.54
19 ^1^A′	9.754	0.14702	3*s*(17*a*^′^) *← n̅*_*O*_/σ_CC_(14*a*^′^) (48%) + π*(5*a*^*″*^) ← π(2*a*^*″*^) (24%) + 4*s*(21*a*^′^) *← n̅*_*O*_/σ_CN_(15*a*^′^) (11%)	9.54(5)	22.87

a See text for details.

b The last decimal of the energy value is given in parentheses for these less-resolved features.

**Table 5:** Proposed Vibrational Assignments of C_2_H_5_NO_2_ Valence and Rydberg Series Converging to the Ionic Electronic Ground (16a′)^−1^ and First (15a′)^−1^ Excited States in the Photon Energy Range 9.0–10.8 (Energies in eV)^a^

energy^b^	assignment	Δ*E* (υ_4_′)	Δ*E* (υ_5_′)
3*s*(17*a*^′^) ← *n̅*_*O*_/σ_CC_(14*a*^′^) + π^*^ (5*a″*) ← π(2*a*^*″*^)			
9.13(7)(s,w)	0_0_^0^		
9.20(1)(s,w)	5_0_^1^		0.064
9.26(3)(s,w)	5_0_^2^		0.062
9.31(9)(s,w)	5_0_^3^		0.056
9.38(2)(s,w)	5_0_^4^		0.063
9.44(6)(w)	5_0_^5^		0.064
			0.062
9.48(3)(w)	3*d*(15*a*′)^−1^		
9.54(5)(b)	4*s*(16*a*′)^−1^		
9.58(9)(b)	5_0_^1^/4_0_^1^	0.106	0.044
9.66(0)(w)	5_0_^2^		0.071
9.71(7)(b)	5_0_^3^/4_0_^2^	0.128	0.057
9.78(2)(s)	5_0_^4^		0.065
9.82(4)(s,w)	5_0_^5^/4_0_^3^/4p (16*a*′)^−1^	0.107	0.042
9.82(4)(s,w)	4p (16*a*′)^−1^		
···	···		
9.94(7)(s,w)	5^*n*^/4_0_^1^	0.123	
9.995	5^*n*+1^		0.048
10.05(6)(s)	5^*n*+2^/4_0_^2^/4*s*(15*a*′)^−1^	0.109	0.061
10.12(5)(s)	5^*n*+3^		0.069
10.16(7)(s)	5^*n*+4^/4_0_^3^	0.111	0.042
10.20(0)(s)	4*d*(15*a*′)^−1^		
10.26(8)(s)	5_0_^1^		0.068
10.38(0)(s)	5p (16*a*′)^−1^		
10.44(5)(b)	5p (16*a*′)^−1^ + 5_0_^1^		0.065
		0.114	0.057

a See text for details.

b (s) shoulder structure; (w) weak feature; (b) broad structure (the last decimal of the energy value is given in parentheses for these less-resolved features);.

We will now discuss in detail each part of these spectra in turn assigning all of the observed features noting the adopted notation *X*_*m*_^*n*^, with *m* and *n* representing the initial and final vibrational states for the vibrational mode (*X*), together with the information provided with the present TD-DFT calculations. Any other assignments involving the vibrational mode (*X*) for which is not possible to determine the vibrational states (*n*, *m*) from the (0 – 0) transition are denoted *X*^*n*^, *X*^*n*+1^, ···

### V.A Nitromethane, CH_3_NO_2_

The major electronic transitions of the photoabsorption bands are assigned to the promotion of an electron from in-plane oxygen lone pair *n̅*_*O*_ (HOMO), *n̅*_*O*_/σ_CN_ (HOMO-1), π_N=O_ (HOMO-2), and π_N=O_ (HOMO-3) to lowest unoccupied molecular orbitals (Table 1 and S1, Figure S5). The VUV spectrum has been measured previously by Walker and Fluendy^1^ and Shastri et al.;^3^ however, the present higher resolution has allowed us to perform a complete vibrational analysis of the fine structure (Tables 2 and 3), while the two lowest-lying absorption bands are broad and structureless.

The lowest absorption band in nitromethane, centered at 4.550 eV and a magnitude of 0.04 Mb (Figure 1), is assigned to the π*(4*a*^″^) ← *n̅*_*O*_/σ_CN_(12*a*^′^),(2^1^*A″* ← *X̃*^1^A′) transition (Table 1), and in good agreement with the 4.5 eV value reported by Walker and Fluendy.^1^ This is also predicted to be a weak transition whose oscillator strength is calculated to be *f*_L_ < 0.0001 (Table 1).

The next structureless but intense absorption band is centered at 6.271 eV (17.74 Mb) and in good agreement with previous work.^1,3^ It is assigned to the π*(4*a*^″^) ← π(3*a*^″^), (2^1^A′ ← *X̃*^1^A′) transition with an oscillator strength of ∼0.1546 (Table 1). The dissociative character of this band yielding NO_2_ has been investigated by photodissociation methods,^38−40^ with electronic and vibrational pre-dissociations as the main mechanisms. In the *C*_2v_ point group, the former has been suggested to occur via a σ* ← σ(^1^*B*_2_,*b*_2_*a*_1_^*^) transition leading to *NO*_2_^*^ which further dissociates into NO (*X̃*) + O, the latter through effective vibrational coupling between NO_2_ and CN modes on the π* ← π potential energy surface, producing NO_2_^**^.^1,38−40^ Regarding the two mechanisms, although in the present experiment we have no information on the spectroscopic nature of the fragments being produced, pre-dissociation can only occur with access at higher energies to an electronic state (e.g., π*) that may cross with an antibonding σ* repulsive state, as long as the nuclear wave packet survives long enough for the system to change its character, resulting in NO_2_ formation. The present calculations in Table 1 do not predict any electronic transition of σ* character close to the π* ← π most intense absorption band. The other mechanism that may prevail, is an efficient vibronic coupling. A close inspection of Figure 2 shows an electronic transition assigned to 3*s*(14*a*^′^) ← *n̅*_*O*_/σ_CN_(12*a*^′^) that overlaps with the high-energy side of the π*(4*a*^″^) ← π(3*a*^″^) transition. The main vibrational modes have been assigned to C–N stretching, *v*_4_^′^(*a*^′^), and NO_2_ symmetric bending, *v*_5_^′^(*a*^′^), the former with several quanta being excited that may couple with the closest electronic state (see Figure S7 and Table S1) as the *R*_CN_ coordinate is stretched from its equilibrium position leading to bond breaking, thus lending strong support to this assumption. Note that such an electronic transition is of Rydberg character, as such one may expect an internal conversion to a valence state that can lead to dissociation within the complex potential energy surfaces involved (see Section VIII.A).

The next electronic transition with its vertical 0_0_^0^ origin is tentatively assigned at 7.382 eV (Figure 2) and is due to the promotion of an electron from the (HOMO-1), 12a′, to a Rydberg state (Table 2), which will be discussed in Section VI. This transition is accompanied by excitation of NO_2_ symmetric stretching, *v*_3_^′^(*a*^′^), C–N stretching, *v*_4_^′^(*a*^′^), NO_2_ symmetric bending, *v*_5_^′^(*a*^′^), and NO_2_ rocking, *v*_8_^′^(*a*^′^) modes (tentative assignments in Table 2), that overlap with the next electronic transition. At 7.66(3) eV we assign the absorption band to a mixed valence-Rydberg character 3*p*_*x*_/σ(16*a*^′^) ← *n̅*_*O*_ (13*a*^′^) + 3*p*_*y*_(15*a*^′^) ← *n̅*_*O*_(13*a*^′^), (2^1^*A*′ ← *X̃*^1^*A*′) with a maximum at 7.92(5) eV and a cross section of 5.98 Mb (Table 1). The vertical value is in good accord with Shastri et al.^3^ although Walker and Fluendy^1^ report it at 7.8 eV. This band shows fine structure, which has been assigned to C–N stretching and NO_2_ symmetric bending modes. For further discussion, see Section VII.

The vertical excitation energies of the next valence, mixed valence-Rydberg, and Rydberg transitions have been assigned at 8.803, 9.450, 10.315, and 10.739 eV. These are labeled in Table 1 π^*^(4*a*^*″*^) ← π_NO_(2*a*^*″*^) + 3*p*_*x*_*/σ*(16*a*^′^) ← *n̅*_*O*_/σ_CN_(12*a*^′^), σ_CN_^*^(17*a*^′^) ← *n̅*_*O*_*/*σ_CN_(12*a*^′^) + 4*s*/4*p*_*y*_(19*a*^′^) ← *n̅*_*O*_(13*a*^′^), 4*s/*5*p*_*z*_(20*a*^′^) ← *n̅*_*O*_/σ_CN_(12*a*^′^) and 4*s*/π*(20*a*^′^) ← *n̅*_*O*_/σ_CN_(12*a*^′^) with cross sections of 16.33, 27.35, 23.63, and 22.32 Mb, respectively. The fine structures in these bands are assigned in Table 3 and will be discussed in Section VII. For the first transition, the valence character is due to the promotion of an electron from (HOMO-3), 2a″, to (LUMO), 4a″, contributing to 70% of the oscillator strength, while for the second transition, (17*a*^′^) ← (12*a*^′^) the antibonding σ* character accounts for 79% (Table 1) which can be indicative of the considerable underlying background signal superimposed on the absorption band. The third transition is due to promotion from (HOMO-1), 12a′, to (LUMO+9), 20a′, where a significant π* character is discernible (see Figure S5). Finally, a reduction of NO_2_ symmetric stretching, *v*_3_^′^(*a*^′^) mode from its value in the ground-state is noted, which agrees with the rather dissociative character of the electronic transitions above 7 eV (see Section VIII).

### V.B Nitroethane, C_2_H_5_NO_2_

The absorption spectrum of nitroethane shows broad structureless bands at 4.450, 6.256, 7.847, 8.271, and 8.725 eV with local maximum cross sections of 0.04, 16.10, 7.76, 11.48, and 18.60 Mb, respectively (Table 4). Due to the absence of any discernible vibrational features, it was not possible to assign the 0_0_^0^ origin of the bands. These bands have been assigned to transitions from the *X̃*^1^*A*^′^ lowest neutral ground-state to valence, mixed valence-Rydberg, and Rydberg states π*(5*a*^*″*^) *← n̅*_*O*_/σ_CN_(15*a*^′^), π*(5*a*^″^) ← π(4*a*^″^), π*(5*a*^″^) *←* n_O_/σ_CH_(3*a*^″^) + 3*s*(17*a*^′^) ← *n̅*_*O*_/σ_CN_(15*a*^′^), *3s*(19*a*^′^) ← *n̅*_*O*_ (16*a*^′^) + 3*s*(18*a*^′^) ← *n̅*_*O*_/σ_CN_(15*a*^′^) and 4*s*/3*p*_*y*_/3*p*_*z*_(20*a*^′^) ← *n̅*_*O*_(16*a*^′^). The lowest absorption band at 4.550 eV is shown in Figure 4 and is attributed to the excitation on an oxygen lone pair electron from the (HOMO-1), 15a′, to the (LUMO) π*(5*a*^″^) orbital (see Figure S6 and Table S2) with a calculated *f*_L_ < 0.0001 (Table 4). The most intense valence π* ← π transitions in the photon energy range 3.7–9.0 eV have oscillator strengths calculated to be *f*_L_ = 0.11079 (6.256 eV) and *f*_L_ = 0.06883, the latter contributing to 70% of the band’s intensity (Table 4). Above 9.0 eV, the mixed valence-Rydberg transitions have been assigned at 9.44(6) and 9.54(5) eV with cross sections of 22.54 and 22.87 Mb. These are mainly due to excitations from (HOMO-4), 14a′, to (LUMO+1), 17a′, and are accompanied by weak vibrational features (Figure 5) which have been assigned in Table 5 to C–N stretching and NO_2_ symmetric bending modes (see Section VII). The transitions with Rydberg character will be discussed in detail in Section VI.

## VI Rydberg Series

The members of a Rydberg series with energy *E_n_* have been fitted with the Rydberg formula: *E*_*n*_*= E*_*i*_*– R*/*(n–* δ)^2^, where *E*_*i*_ is the ionization energy, *n* is the principal quantum number of the Rydberg orbital of energy *E_n_*, *R* is one Rydberg (13.61 eV), and δ is the quantum defect resulting from the penetration of the Rydberg orbital into the core. Due to lower resolution in previous experiments and limited range of the photoabsorption spectra, new vibrational features have been assigned in the Rydberg region, with only a few being previously reported for nitromethane,^1,3^ while, to the authors’ knowledge, no information for nitroethane is available in the literature. Therefore, in the following discussion, we present a detailed analysis of the Rydberg series (Tables 6 and 7) and their fine structure assignments in Section VII and Tables 2, 3, and 5.

**Table 6:** Energy Values (eV), Quantum Defects (δ), and Assignments of the Rydberg Series Converging to Ionic Electronic Ground (13a′)^−1^*X̅*^2^*A*^′^, and First (12a′)^−1^*A*^~2^*A*′ Excited States of CH_3_NO_2_^a^ Compared with Previous Work^b^

(IE_1_)_ad_ = 11.07 eV (13*a*′)^−1^				(IE_2_)_ad_ = 11.73 eV (12*a*′)^−1^			
*E*_n_	δ	assignment	*E*_n_^3^	*E*_n_	δ	assignment	*E*_n_^3^
*(ns ← 13a′)*				*(ns ← 12a′)*			
7.637	1.01	3s	7.531	8.38(3)(s)	0.98	3s	8.257
9.552	1.01	4s	9.541	10.251	0.97	4s	10.247
10.251	0.92	5s	10.170				
10.54(3)(b)	0.92	6s	10.493				
*(np ← 13a′)*				*(np ← 12a′)*			
8.420	0.73	3p	8.732	9.08(3)(s)	0.73	3p	9.126
9.77(0)(s)	0.76	4p	9.761	10.445	0.75	4p	10.439
10.315	0.75	5p	10.309				
10.583	0.71	6p	10.573				
8.803	0.55	3p′	8.792	9.450	0.56	3p′	9.315
9.91(1)(s)	0.57	4p′	9.806	10.583	0.55	4p′	10.493
10.39(3)(w)	0.52	5p′	10.347				
10.629	0.45	6p′	10.628				
*(nd ← 13a′)*				*(np ← 12a′)*			
9.379	0.16	3d	9.518	10.01(5)(s)	0.18	3d	10.066
10.14(6)(s)	0.16	4d					
10.498	0.12	5d					
10.66(5)(s)	0.20	6d					

a (s) shoulder structure; (b) broad structure (the last decimal of the energy value is given in parentheses for these less-resolved features).

b See text for details.

**Table 7:** Energy Values (eV), Quantum Defects (δ), and Assignments of the Rydberg Series Converging to Ionic Electronic Ground (16a′)^−1^*X̃*^2^*A*′, and First (15a′)^−1^*Ã*^2^*A′* Excited States of C_2_H_5_NO_2_^a^,^b^

(IE_1_)_v_ = 11.08 eV (16*a*′)^−1^			(IE_2_)_v_ = 11.51 eV (15*a*′)^−1^		
*E*_n_	δ	assignment	*E*_n_	δ	assignment
*(ns ← 16a′)*			*(ns ← 15a′)*		
7.54(2)(s,w)	1.04	3s	8.26(6)(b)	0.95	3s
9.54(5)(b)	1.02	4s	10.05(6)(s)	0.94	4s
10.22(1)(s)	1.02	5s	10.65(2)(s,w)	1.02	5s
10.53(8)(s,w)	0.99	6s			
10.77(1)(s)	0.93	7s			
*(np ← 16a′)*			*(np ← 15a′)*		
8.52(1)(s,w)	0.69	3p	8.86(9)(s,w)	0.73	3p
9.82(4)(s,w)	0.71	4p	10.22(1)(s)	0.75	4p
10.34(1)(s,w)/10.38(0)(s)	0.71/0.59	5p	10.77(1)(s)	0.71	5p
10.59(7)(w)	0.69	6p			
*(nd ← 16a′)*			*(nd ← 15a′)*		
9.48(3)(w)	0.08	3d	9.79(7)(s,w)	0.18	3d
10.20(0)(s)	0.07	4d	10.60(6)(b,w)	0.12	4d
10.52(1)(s,w)	0.07	5d			
10.71(1)(s,w)	–0.07	6d			

a (s) shoulder structure; (w) weak feature; (b) broad structure (the last decimal of the energy value is given in parentheses for these less-resolved features).

b See text for details.

### VI.A Nitromethane, CH_3_NO_2_

The VUV photoabsorption cross section above 7.5 eV consists of a series of sharp peaks assigned as *n*s, *n*p, *n*p′, and *n*d Rydberg states (Figure 1) converging to the ionic electronic ground (13a′)^−1^*X̃*^2^*A*^′^ and first (12a′)^−1^*Ã*^2^*A*^′^ excited states, with adiabatic values of 11.07 and 11.73 eV. Some of the members in the Rydberg series are in good agreement with the values reported by Walker and Fluendy,^1^ and Shastri et al.^3^ but we have been able to assign *n* > 3 members of the *n*d(13a′)^−1^ series (Table 6).

The first member of an *n*s series is found to lie at 7.637 eV (Table 6), is assigned to the (3*s* ← (13*a*^′^)) excitation, with a quantum defect δ = 1.01 (Table 6), and is coincident with a component of a vibrational excitation pattern (Section VII). The 4s term appears at 9.552 eV with a quantum defect of 1.01, also showing a modest associated vibrational excitation. The calculated vertical excitation energy is assigned at 9.702 eV (Table 1) and is due to the 4*s*(19*a*^′^) ← *n̅*_*O*_(13*a*^′^) transition with the contribution of a 4p Rydberg series. Subsequent Rydberg features are observed up to *n* = 6.

The first members of the two n*p* (*np* ← *13a′*) and (*np*′ ← *13a′*) series are associated with absorption features at 8.420 and 8.803 eV (δ = 0.73 and 0.55, respectively) (Table 5), the latter has also been assigned to contribute to a valence π*(4*a*^″^) ← π_NO_(2*a*^″^) transition (Table 1). The *n*d series extends up to *n* = 8 with average quantum defects of δ = 0.74 and 0.52, respectively. The members *n* = 3, 4, and 6 of the *n*d series are also part of a vibrational pattern in this region of the absorption spectrum.

Our assignments also report the presence of one n*d* (*nd* ← *13a′*) series, with the *n* = 3 feature at 9.379 eV (δ = 0.16) (Table 5), while other transitions to higher members of the Rydberg series up to *n* = 6 are also discernible. The first member of the *n*d series and the feature at 10.66(5) eV are coupled with C–N stretching and NO_2_ symmetric bending modes (Section VII).

The Rydberg series converging to the ionic electronic first excited state are listed in Table 5 and have been assigned to the (*ns,np,np*′*,nd* ← 12*a*^′^) transitions. The first members of the *ns,np,np*′*,nd* series are associated with features at 8.38(3) eV (δ = 0.98), 9.08(3) eV (δ = 0.73), 9.450 eV (δ = 0.56), and 10.01(5) eV (δ = 0.18) (Table 5). The calculated vertical excitation energy of 3*s* is at 7.510 eV (*f*_L_ ≈ 0.0363) (Table 1), while Shastri et al.^3^ report it at 7.845 eV (*f*_L_ ≈ 0.0354). The 10.251 and 10.583 eV features assigned to 4s and 4p′ can also be ascribed to 5s(13*a*′)^−1^ and 6p(13*a*′)^−1^, coupled with vibrational excitation. Tentative assignments of these series have only been made for *n* = 4 because higher members lie outside the photon energy range investigated.

### VI.B Nitroethane, C_2_H_5_NO_2_

The first member of *n*s Rydberg transition is assigned to (4*s* ← (16*a*^′^)) at 7.54(2) eV and with a quantum defect δ = 1.04, while other transitions to higher-order Rydberg members *n* = 7, are also reported in Table 7. Note that the shape of the absorption band where the 4s member is assigned can also be due to a valence character (24%) (see Table 4), and the slightly larger value of that quantum defect for the 4s member is attributed to the influence of the vibrational excitation observed in this region of the absorption spectrum.

The lowest-lying member of the n*p* (*np* ← 1*6a′*) series is associated with the absorption feature at 8.52(1) eV (δ = 0.69) (Table 7). The relative increase in the value of the quantum defect for the 4p member of the Rydberg series (δ = 0.71) can be attributed to vibrational features, as discussed in Section VII.

The assignments in Table 7 also report the presence of one n*d* (*nd* ← 1*6a′*) series, with its first member, the *n* = 3, at 9.48(3) eV (δ = 0.08), while other members have been assigned up to *n* = 6.

Three Rydberg series converging to the ionic electronic first excited state are listed in Table 7 and have been assigned to the (*ns,np,nd* ← 15*a*^′^) transitions. The lowest-lying members of the *ns,np,nd* series are associated with features at 8.26(6) eV (δ = 0.95), 8.86(9) eV (δ = 0.73), and 9.79(7) eV (δ = 0.18). We note that features at 10.22(1) and 10.77(1) eV assigned to 4p(15*a*′)^−1^ and 5p(15*a*′)^−1^ are also due to excitations from the ground state to 5s(16*a*′)^−1^ and 7s(16*a*′)^−1^ members of Rydberg series (Table 7). Tentative assignments of the (*ns,np,nd* ← 15*a*^′^) series have only been made for *n* = 5 because members for *n* > 5 lie outside the photon energy range.

## VII Vibrational Excitation Coupled with Rydberg Series

### VII.A Nitromethane, CH_3_NO_2_

Vibrational excitation associated with several of the Rydberg series is presented in detail in Tables 2 and 3. However, to avoid congestion in the Rydberg series, we have not labeled these in the figures but only indicated their energy positions by vertical bars in the series (Figure 3). The three modes being excited are mainly those already reported for the valence excitation in the 7.3–8.3 eV energy region, they correspond to NO_2_ symmetric stretching, *v*_3_^′^(*a*^′^), C–N stretching, *v*_4_^′^(*a*^′^), NO_2_ symmetric bending, *v*_5_^′^(*a*^′^) and NO_2_ rocking, *v*_8_^′^(*a*^′^). The excitation of these modes is characterized by average energies of 0.157, 0.098, 0.071, and 0.045 eV, respectively, with ground-state values of *v*_3_^′^(*a*^′^) = 0.171 eV, *v*_4_^′^(*a*^′^) = 0.114 eV, *v*_5_^′^(*a*^′^) = 0.081 eV and *v*_8_^′^(*a*^′^) = 0.059 eV.^8,9^ Combination bands of these modes and progressions, involving the NO_2_ symmetric bending, *v*_5_^′^(*a*^′^) and the NO_2_ rocking, *v*_8_^′^(*a*^′^), have also been assigned. The normal mode description of vibrations is relevant for the lowest-lying excitations, with the possibility of Fermi resonances (Table 2).

### VII.B Nitroethane, C_2_H_5_NO_2_

The high-resolution VUV spectrum shows the presence of some diffuse structures (see Figures 4 and 5) mainly above 9.0 eV, which we have tentatively assigned as vibrational excitations (Table 5). From the mean energy separation of 0.114 and 0.059 eV, the structure may be attributed to excitation of C–N stretching, *v*_4_^′^(*a*^′^) and NO_2_ symmetric bending, *v*_5_^′^(*a*^′^) modes, with ground-state values of *v*_4_^′^(*a*^′^) = 0.109 eV and *v*_5_^′^(*a*^′^) = 0.077 eV. The fine structure involving the Rydberg series converging to the (16*a*′)^−1^ and (15*a*′)^−1^ states have been marked in Figure 5, with the assignments only included in Table 5, to avoid congestion of the figure. Combination bands of these modes and progressions involving the NO_2_ symmetric bending mode *v*_5_^′^(*a*^′^) are also assigned in Table 5.

## VIII Potential Energy Curves along the C–N Coordinate

Potential energy curves (PECs) along the C–N bond have been obtained at the TD-DFT/B3LYP/aug-cc-pVDZ level of theory for nitromethane and nitroethane in the *C*_s_ symmetry group. The molecules’ other geometric parameters were kept frozen at their equilibrium values. The lowest-lying excited A′ and A″ states in Figures S7 and 6, for nitromethane and nitroethane, together with their characters at Δ*R*_C–N_ = 0.6 Å, are plotted for the *eclipsed* conformers, while those for the *staggered* conformers are not shown since no appreciable relative differences were found.

**Figure 6:**
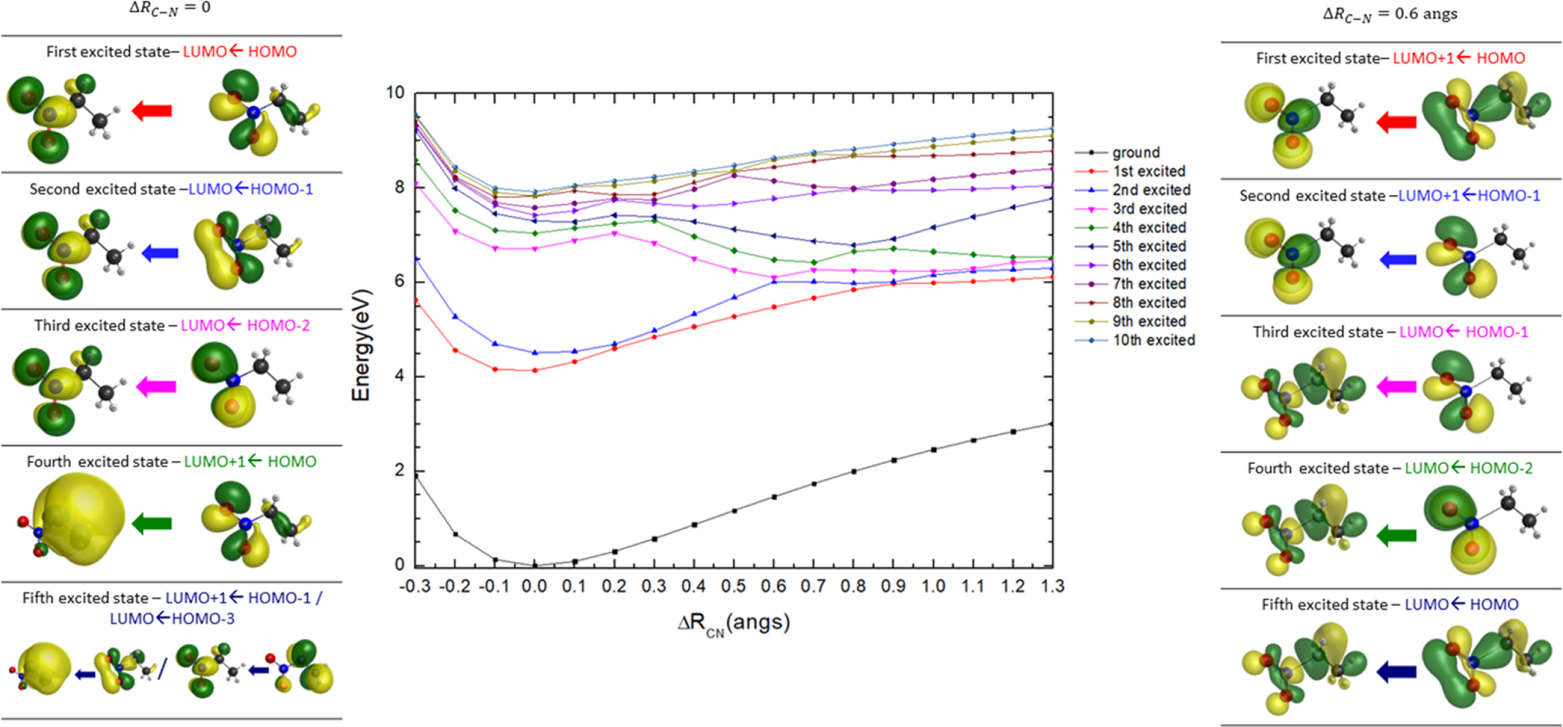
PECs for the ground and low-lying excited singlet states of C_2_H_5_NO_2_, plotted as a function of the *R*_C–N_ coordinate, and calculated at the TD-DFT/B3LYP/aug-cc-pVDZ level of theory in the *C*_s_ symmetry group. See text for details.

### VIII.A Nitromethane, CH_3_NO_2_

Ultrafast photodissociation dynamics studies of Nelson et al.^15^ at 266 nm (4.661 eV) have revealed that NO_2_ dissociation was found to be relatively fast (81 fs) compared to the slower isomerization process (452 fs) that can occur via recombination of the CH_3_ radical and NO_2_ yielding CH_3_ONO. Additionally, Nelson et al.^15^ have reported a calculated NO_2_ abstraction with a quantum yield of *φ*_Cl_(CH_3_NO_2_) = 0.24.

The first two excited states show a bonding potential well even when the Δ*R*_C–N_ coordinate is increased, where dissociation can only be attained for Δ*R*_C–N_ > 0.6 Å. This may explain the absence of a dissociative character in the absorption bands peaking at 4.550 and 6.271 eV (Figure 1). This is in accordance with previous work.^3^ However, as the C–N bond is stretched to 0.6 Å, the rather weak C···N character of HOMO-1 and HOMO-2 bonds, show the imminent dissociative character involving any electron promotion to the LUMO and LUMO+1 for the four lowest-lying excited states (Figure S7, right).

The behavior of the lowest-lying π*(4*a*^*″*^) *← n̅*_*O*_/σ_CN_(12*a*^′^) transition is in agreement with the ultrafast dissociation dynamics of nitromethane through C–N bond excision (81 fs), followed by a fast isomerization and subsequent rebinding of CH_3_ and NO_2_.^15^ However, if dissociation may be operative in this energy region, then the multidimensional landscape of the reaction coordinates available in nitromethane may follow a different route which is not via the C–N coordinate. Yue et al.^41^ have reported photodissociation studies at 266 nm (4.661 eV) of nascent OH being produced vibrationally cold. Note that Shastri et al.^3^ have also reported PECs as a function of the NO bond length to discuss the pathway yielding CH_3_NO + O, as another mechanism in the photodissociation dynamics of nitromethane. For further detailed description of the nuclear dynamics that may govern the (pre)dissociative character of the low-lying excited states, see ref (3) and references therein.

A close inspection of Figure S7 shows that above 7 eV, the rather dissociative character observed in the photoabsorption bands (Figure 1) is due to a background contribution, thus shifting the absorption features from the baseline of the spectrum. The dissociative character of these transitions can be explained by the quasi-degenerate nature of the potential energy curves at given Δ*R*_C–N_ values, where the nuclear wave packet can adiabatically evolve “down the hill”, either correlating with the asymptotic limits of the second or the third excited states. It is interesting to note that internal conversion from Rydberg to valence character may be operative at those energies, which is clearly depicted in the molecular orbital representations from the left to the right panels of Figure S7 (e.g., the fourth and the second excited states). This mechanism is responsible for vibronic coupling that can hold for the close-lying overlap between, e.g., *3s*(14*a*^′^) ← *n̅*_*O*_/σ_CN_(12*a*^′^) and π*(4*a*^*″*^) *←* π(3*a*^″^) transitions (see Figure 2), where fine structure has been assigned to C–N stretching, *v*_4_^′^(*a*^′^) and NO_2_ symmetric bending, *v*_5_^′^(*a*^′^) modes. Note that the underlying dynamics governing the internal conversion is a rather complex process given the different reaction coordinates involved. However, the rather bonding nature of the excited electronic states may render some pre-dissociative character in the absorption spectrum, which becomes more noticeable for higher-lying excited states above 9 eV, where at smaller relative Δ*R*_C–N_ values (0.4 Å) these states can already cross with others correlating with their asymptotic limits yielding bond breaking. It is commonly accepted that the theoretical calculation methodology employed here does not provide an accurate description of the higher-lying excited states. However, these have been obtained and those PECs plotted in Figure S7 only serve to give a qualitative description of the reaction coordinate as it is stretched from its equilibrium geometry. This molecular bond breaking description is certainly more intricate and may involve rather complex nuclear dynamics in the multidimensional potential energy surfaces.

### VIII.B Nitroethane, C_2_H_5_NO_2_

The two lowest-lying excited states show a rather similar behavior as noted in nitromethane, i.e., the bonding nature of the PECs occurs even at larger Δ*R*_C–N_ values (∼0.6 Å) meaning that the absorption features in the spectrum are due to π*(5*a*^″^) *← n̅*_*O*_/σ_CN_(15*a*^′^) and π*(*5a*^*″*^) ← π(4*a*^″^) as noted before. Additionally, photodissociation studies at 266 nm (4.661 eV) reported vibrationally cold OH and NO radicals,^41,42^ thus meaning that the nuclear dynamics of the lowest-lying electronic states of nitroethane are complex mechanisms within the accessible potential energy surfaces as a function of the different degrees of freedom. However, above 8 eV the considerable relevant crossing between the different PECs may be responsible for the efficient dissociative character of these states, some correlating with the asymptotic limit at ∼6.5 eV and at higher energies at ∼8 eV. Also, relevant above 7 eV is the conversion from Rydberg (e.g., fourth excited state) to valence character (e.g., third excited state) as the reaction coordinate changes within the adiabatic description of the potential energy curves involved. It is worth mentioning that from a close inspection of Figure 6, nitroethane PECs show for the higher-lying excited states a more bound (deeper potential energy wells) character than in nitromethane. However, the crossings between different electronic states are noted at lower Δ*R*_C–N_ values (ca. 0.2–0.3 Å) than in nitromethane (∼0.4 Å), thus resulting in less extensive fine structure and so less relevant pre-dissociative character but rather more relevant bond breaking. The MOs with electron densities up to the fifth excited electronic state show the dissociative character of the nuclear dynamics as a function of the C–N stretching coordinate.

**Figure 7:**
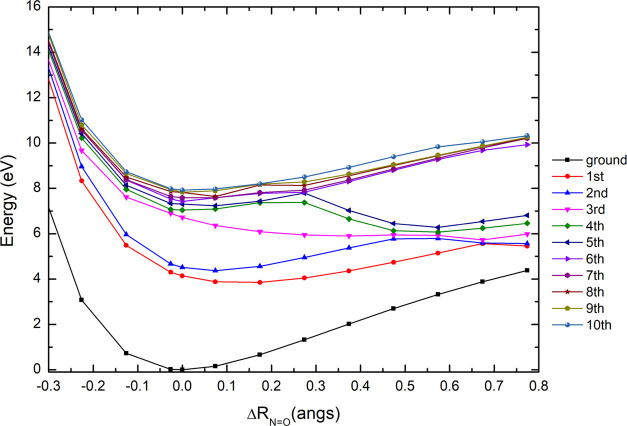
PECs for the ground and low-lying excited singlet states of C_2_H_5_NO_2_, plotted as a function of the *R*_NO_ coordinate, and calculated at the TD-DFT/B3LYP/aug-cc-pVDZ level of theory in the *C*_s_ symmetry group. See text for details.

We are not aware of any previous photodissociation studies along the NO coordinate, yet studies by Li et al.^42^ of the photolysis of nitroethane at 266 nm (4.661 eV) report mainly the C_2_H_5_O + NO reaction, noting that the competition among the different energetically accessible channels may also lead to C_2_H_5_ + NO_2_ → C_2_H_5_ + O + NO. Nonetheless, potential energy curves for the eight lowest-lying singlet electronic states above the neutral ground-state have been obtained as a function of the NO stretching mode (Figure 7). The significant dissociative character attained through access to the third electronic state at ∼7.5 eV may also correlate in the asymptotic limit with higher-lying states through efficient curve crossing. At higher energies, although a few potential energy wells are quite shallow with modest barriers, relevant crossing at ∼0.3 Å, may allow an efficient dissociative process (via pre-dissociation). The nitroethane spectrum in Figure 4 shows that above 8 eV, the photoabsorption features are quite shifted from the baseline signal, which can be indicative of the dissociative and/or pre-dissociative nature of those transitions.

## IX Absolute Photoabsorption Cross-Sections and Atmospheric Photolysis

Using the Beer–Lambert law (Section II) we have derived absolute photoabsorption cross sections over the energy range 3.7–10.8 eV. The π* ← π absorption bands in nitromethane were found to have maximum cross sections of 0.04 and 16.10 Mb at 4.550 and 6.256 eV, respectively, with identical values reported in the earlier photoabsorption studies of Walker and Fluendy.^1^ As far as nitroethane is concerned, the π* ← *n̅*_*O*_ transition at 4.476 eV (277 nm) with a maximum cross section of 0.04 Mb (4.476 eV) is in excellent agreement with the available MPI-Mainz UV/VIS Spectral Atlas data^43^ while Goodeve^24^ reported a value of ∼0.06 Mb. Using the present cross section values, we can model the atmospheric destruction of CH_3_NO_2_ and C_2_H_5_NO_2_ by ultraviolet photolysis as a function of altitude in the Earth’s atmosphere from sea level up to the limit of the stratopause (50 km). Details of the program are presented in a previous publication by Limão-Vieira^44^ and co-workers. Photolysis rates at a given wavelength were calculated as the product of the solar actinic flux,^45^ and the molecular photoabsorption cross section at 1 km altitude steps from the surface up to the stratopause. At each altitude, a total photolysis rate may then be calculated by summing over the individual photolysis rates for that altitude. The reciprocal of the total photolysis rate for a given altitude gives the local photolysis lifetime at that altitude, i.e., the time taken for the molecule to photodissociate at that altitude assuming the solar flux remains constant. The quantum yield for NO_2_ elimination from nitromethane is taken to be 0.24 at 266 nm, from the work of Nelson et al.,^15^ and an identical value is assumed for nitroethane due to lack of any information in the literature and for the similar magnitude of the lowest-lying absorption bands of both chemical compounds. Computed photolysis lifetimes of less than 1 sunlit day were calculated at all altitudes above ground level, therefore indicating that the nitromethane and nitroethane molecules can be efficiently photolyzed at these altitudes. Liu and co-workers^18^ reported a complete study of the gas-phase kinetics for the CH_3_NO_2_ + OH and C_2_H_5_NO_2_ + OH reactions, as a function of the temperature (240–400 K), reporting rate values of *k*_OH_ (298 K) = (1.58 ± 0.09) × 10^–14^ cm^3^ molecule^–1^ s^–1^ and *k*_OH_ (298 K) = (7.22 ± 0.82) × 10^–14^ cm^3^ molecule^–1^ s^–1^, respectively. As far as we are aware, there is no comprehensive study on the lifetimes for the reactions of nitromethane and nitroethane with ^●^OH radicals, or any other oxidation processes, e.g., reactions with NO_*x*_ pollutants and their products produced in fossil fuel combustion, to assess the role of such processes as the main sink mechanism for these molecules.

## X Conclusions

We have measured high-resolution VUV spectra of nitromethane and nitroethane over the energy range between 3.7 and 10.8 eV using synchrotron radiation. Photoabsorption bands attributed to valence, mixed valence-Rydberg, and Rydberg transitions are observed in the spectra. All of these bands have been identified and compared with previous work when available, with several new structures being observed and assigned for the first time. The electronic state spectroscopy of nitroethane has allowed the assignment, for the first time, of fine structure in the spectra, which are due to the contributions of C–N stretching and NO_2_ symmetric bending modes. The assignment of the electronic transitions was performed with the aid of TD-DFT calculations on the vertical excitation energies and oscillator strengths. The absolute photoabsorption cross sections have also been measured and were used to derive photolysis rates of both chemical compounds in the terrestrial atmosphere from the seal level up to the stratosphere, indicating that solar photolysis is expected to be a strong sink for these molecules at all altitudes above sea level. Reactions with ^•^OH radicals seem to be relevant; however, comprehensive studies of the gas-phase lifetimes of CH_3_NO_2_ and C_2_H_5_NO_2_ with hydroxyl in the Earth’s atmosphere are needed to properly assess the role of such reactions. Further work on the atmospheric chemistry of both compounds is needed to quantify their role in global warming and ozone depletion. Potential energy curves as a function of the C–N coordinate, for the lowest-lying excited A′ and A″ states, were obtained at the TD-DFT/B3LYP/aug-cc-pVDZ level of theory. These have shown that the pre-dissociative character along the C–N reaction coordinate is more efficient in nitromethane than in nitroethane within the accessible Franck–Condon region of the electronic transitions.

## Supporting Information Available

Ground-state geometries of nitromethane and nitroethane conformers; ionic electronic ground-state geometries of nitromethane and nitroethane; representation of molecular orbitals of nitromethane and nitroethane; and potential energy curves for the lowest-lying electronic states of CH_3_NO_2_ as a function of the C–N coordinate (PDF)

## References

[ref1] Walker I. C.; Fluendy M. A. D. Spectroscopy and Dynamics of Nitromethane (CH3NO2) and Its Anionic States. Int. J. Mass Spectrom. 2001, 205, 171–182. 10.1016/S1387-3806(00)00319-5.

[ref2] Lord-Garcia J. Nitromethane. In Encyclopedia of Toxicology, 3rd ed.; Academic Press, 2014; pp 573–574.

[ref3] Shastri A.; Das A. K.; Sunanda K.; Rajasekhar B. N. Electronic States of Nitromethane: Experimental and Theoretical Studies. J. Quant. Spectrosc. Radiat. Transf. 2021, 276, 107933 10.1016/j.jqsrt.2021.107933.

[ref4] McAllister T. Electron Impact Excitation Spectra in an Ion Cyclotron Resonance Mass Spectrometer. J. Chem. Phys. 1972, 57, 3353–3355. 10.1063/1.1678766.

[ref5] Goebbert D. J.; Pichugin K.; Sanov A. Low-Lying Electronic States of CH3NO2 via Photoelectron Imaging of the Nitromethane Anion. J. Chem. Phys. 2009, 131, 164308 10.1063/1.3256233. 19894948

[ref6] Flicker W. M.; Mosher O. A.; Kuppermann A. Variable Angle Electron-Impact Excitation of Nitromethane. J. Chem. Phys. 1980, 72, 2788–2794. 10.1063/1.439427.

[ref7] Linnett J. W.; Avery W. H. Infra-Red and Raman Spectra of Polyatomic Molecules. IV. Allene. J. Chem. Phys. 1938, 6, 686–691. 10.1063/1.1750152.

[ref8] Smith D. C.; Pan C. Y.; Nielsen J. R. Vibrational Spectra of the Four Lowest Nitroparaffins. J. Chem. Phys. 1950, 18, 706–712. 10.1063/1.1747732.

[ref9] Jones W. J.; Sheppard N. The Gas-Phase Infrared Spectra of Nitromethane and Methyl Boron Difluoride; Fine Structure Caused by Internal Rotation. Proc. R. Soc. London, Ser. A 1968, 304, 135–155. 10.1098/rspa.1968.0078.

[ref10] Courtecuisse S.; Cansell F.; Fabre D.; Petitet J. P. Comparative Raman Spectroscopy of Nitromethane-H3, Nitromethane-D3, and Nitroethane up to 20 GPa. J. Chem. Phys. 1998, 108, 7350–7355. 10.1063/1.476154.

[ref11] Kobayashi T.; Nagakura S. Photoelectron Spectra of Nitro-Compounds. Chem. Lett. 1972, 1, 903–907. 10.1246/cl.1972.903.

[ref12] Rabalais J. W. Photoelectron Spectroscopic Investigation of the Electronic Structure of Nitromethane and Nitrobenzene. J. Chem. Phys. 1972, 57, 960–967. 10.1063/1.1678346.

[ref13] Rao C. N. R. Photoelectron Spectra of C-Nitro & N-Nitro Compounds. Indian J. Chem. 1976, 14, 147–149.

[ref14] Mok C. Y.; Chin W. S.; Huang H. H. He (I) and He (II) Photoelectron Spectra of Simple Nitroalkanes. J. Electron Spectrosc. Relat. Phenom. 1991, 57, 213–222. 10.1016/0368-2048(91)85025-O.

[ref15] Nelson T.; Bjorgaard J.; Greenfield M.; Bolme C.; Brown K.; McGrane S.; Scharff R. J.; Tretiak S. Ultrafast Photodissociation Dynamics of Nitromethane. J. Phys. Chem. A 2016, 120, 519–526. 10.1021/acs.jpca.5b09776. 26735907

[ref16] Rodríguez J. D.; González M. G.; Rubio-Lago L.; Bañares L.; Samartzis P. C.; Kitsopoulos T. N. Stereodynamics of the Photodissociation of Nitromethane at 193 Nm: Unravelling the Dissociation Mechanism. J. Phys. Chem. A 2013, 117, 8175–8183. 10.1021/jp403272x. 23713854

[ref17] Campbell I. M.; Goodman K. Rate Constants for Reactions of Hydroxyl Radicals with Nitromethane and Methyl Nitrite Vapours at 292 K. Chem. Phys. Lett. 1975, 36, 382–384. 10.1016/0009-2614(75)80262-4.

[ref18] Liu R.; Huie R. E.; Kurylo M. J.; Nielsen O. J. The Gas Phase Reactions of Hydroxyl Radicals with a Series of Nitroalkanes over the Temperature Range 240-400 K. Chem. Phys. Lett. 1990, 167, 519–523. 10.1016/0009-2614(90)85462-L.

[ref19] Murrell J. N.; Vidal B.; Guest M. F. Structure and Electronic Properties of the Nitromethyl Anion, Nitromethane and Aci-Nitromethane. J. Chem. Soc., Faraday Trans. 2 1975, 71, 1577–1582. 10.1039/f29757101577.

[ref20] Lopes A. R.; Sanchez S. d’A.; Bettega M. H. F. Elastic Scattering of Low-Energy Electrons by Nitromethane. Phys. Rev. A 2011, 83, 062713 10.1103/PhysRevA.83.062713.

[ref21] Antunes R.; Almeida D.; Martins G.; Mason N. J.; Garcia G.; Maneira M. J. P.; Nunes Y.; Limão-Vieira P. Negative Ion Formation in Potassium–Nitromethane Collisions. Phys. Chem. Chem. Phys. 2010, 12, 12513–12519. 10.1039/c004467a. 20721400

[ref22] Alizadeh E.; Ferreira da Silva F.; Zappa F.; Mauracher A.; Probst M.; Denifl S.; Bacher A.; Märk T. D.; Limão-Vieira P.; Scheier P. Dissociative Electron Attachment to Nitromethane. Int. J. Mass Spectrom. 2008, 271, 15–21. 10.1016/j.ijms.2007.11.004.

[ref23] Shafiee A.; Khoobi M. Nitroethane. In Encyclopedia of Toxicology, 3rd ed.; Academic Press, 2014; pp 543–547.

[ref24] Goodeve J. W. The Absorption Spectra of Ethyl Nitrate, Ethyl Nitrite, and Nitroethane. Trans. Faraday Soc. 1934, 30, 504–508. 10.1039/tf9343000504.

[ref25] Palmer M. H.; Ridley T.; Hoffmann S. V.; Jones N. C.; Coreno M.; De Simone M.; Grazioli C.; Biczysko M.; Baiardi A.; Limão-Vieira P. Interpretation of the Vacuum Ultraviolet Photoabsorption Spectrum of Iodobenzene by Ab Initio Computations. J. Chem. Phys. 2015, 142, 134302 10.1063/1.4916121. 25854238

[ref26] Eden S.; Limão-Vieira P.; Hoffmann S. V.; Mason N. J. VUV Photoabsorption in CF3X (X = Cl, Br, I) Fluoro-Alkanes. Chem. Phys. 2006, 323, 313–333. 10.1016/j.chemphys.2005.09.040.

[ref27] Barca G. M. J.; Bertoni C.; Carrington L.; Datta D.; De Silva N.; Deustua J. E.; Fedorov D. G.; Gour J. R.; Gunina A. O.; Guidez E.; et al. Recent Developments in the General Atomic and Molecular Electronic Structure System. J. Chem. Phys. 2020, 152, 154102 10.1063/5.0005188. 32321259

[ref28] Bauernschmitt R.; Ahlrichs R. Treatment of Electronic Excitations within the Adiabatic Approximation of Time Dependent Density Functional Theory. Chem. Phys. Lett. 1996, 256, 454–464. 10.1016/0009-2614(96)00440-X.

[ref29] Casida M. E. Time-Dependent Density-Functional Theory for Molecules and Molecular Solids. J. Mol. Struct.: THEOCHEM 2009, 914, 3–18. 10.1016/j.theochem.2009.08.018.

[ref30] Tannenbaum E.; Myers R. J.; Gwinn W. D. Microwave Spectra, Dipole Moment, and Barrier to Internal Rotation of CH3NO2 and CD3NO2. J. Chem. Phys. 1956, 25, 42–47. 10.1063/1.1742845.

[ref31] Tannenbaum E.; Johnson R. D.; Myers R. J.; Gwinn W. D. Microwave Spectrum and Barrier to Internal Rotation of Nitromethane. J. Chem. Phys. 1954, 22, 949 10.1063/1.1740230.

[ref32] McKee M. L. Ab Initio and MNDO Study of Nitromethane and the Nitromethyl Radical. J. Am. Chem. Soc. 1985, 107, 1900–1904. 10.1021/ja00293a017.

[ref33] Cornaton Y.; Ringholm M.; Louant O.; Ruud K. Analytic Calculations of Anharmonic Infrared and Raman Vibrational Spectra. Phys. Chem. Chem. Phys. 2016, 18, 4201–4215. 10.1039/C5CP06657C. 26784673 PMC5063043

[ref34] Gorse D.; Cavagnat D.; Pesquer M.; Lapouge C. Theoretical and Spectroscopic Study of Asymmetric Methyl Rotor Dynamics in Gaseous Partially Deuterated Nitromethanes. J. Phys. Chem. A 1993, 97, 4262–4269. 10.1021/j100119a005.

[ref35] Brakaspathy R.; Jothi A.; Singh S. Determination of Force Fields for Two Conformers of Nitromethane by CNDO/Force Method. Pramana - J. Phys. 1985, 25, 201–209. 10.1007/BF02847660.

[ref36] Mezey P. G.; Kresge A. J.; Csizmadia I. G. A Theoretical Study on The stereochemistry and Protonation of -:CH2-NO2. Can. J. Chem. 1976, 54, 2526–2533. 10.1139/v76-358.

[ref37] Vicente A.; Antunes R.; Almeida D.; Franco I. J. A.; Hoffmann S. V.; Mason N. J.; Eden S.; Duflot D.; Canneaux S.; Delwiche J.; et al. Photoabsorption Measurements and Theoretical Calculations of the Electronic State Spectroscopy of Propionic, Butyric, and Valeric Acids. Phys. Chem. Chem. Phys. 2009, 11, 5729–5741. 10.1039/b823500g. 19842491

[ref38] Moss D. B.; Trentelman K. A.; Houston P. L. 193 nm Photodissociation Dynamics of Nitromethane. J. Chem. Phys. 1992, 96, 237–247. 10.1063/1.462510.

[ref39] Butler L. J.; Krajnovich D.; Lee Y. T.; et al. The Photodissociation of Nitromethane at 193 Nm. J. Chem. Phys. 1983, 79, 1708–1722. 10.1063/1.446015.

[ref40] Lao K. Q.; Jensen E.; Kash P. W.; Butler L. J. Polarized Emission Spectroscopy of Photodissociating Nitromethane at 200 and 218 Nm. J. Chem. Phys. 1990, 93, 3958–3969. 10.1063/1.458781.

[ref41] Yue X. F.; Sun J. L.; Wei Q.; Yin H. M.; Han K. L. Photodissociation Dynamics of Nitromethane and Nitroethane at 266 Nm. Chin. J. Chem. Phys. 2007, 20, 401–406. 10.1088/1674-0068/20/04/401-406.

[ref42] Li Y.; Sun J.; Han K.; He G.; Li Z. The Dynamics of NO Radical Formation in the UV 266 Nm Photodissociation of Nitroethane. Chem. Phys. Lett. 2006, 421, 232–236. 10.1016/j.cplett.2006.01.055.

[ref43] Keller-Rudek H.; Moortgat G. K.; Sander R.; Sörensen R. The MPI-Mainz UV/VIS Spectral Atlas of Gaseous Molecules of Atmospheric Interest. Earth Syst. Sci. Data 2013, 5, 365–373. 10.5194/essd-5-365-2013.

[ref44] Limão Vieira P.; Eden S.; Kendall P. A.; Mason N. J.; Hoffmann S. V. VUV Photo-Absorption Cross-Section for CCl2F2. Chem. Phys. Lett. 2002, 364, 535–541. 10.1016/S0009-2614(02)01304-0.

[ref45] Chemical Kinetics and Photochemical Data for Use in Stratospheric Modelling, Evaluation Number 12, NASA, Jet Propulsion Laboratory, JPL, Publication 97-4, January 15; 1997.

