# Supplementary material for: Electronic State Spectroscopy of Nitromethane and Nitroethane
Source: J Phys Chem A. 2023 Feb 2;127(6):1445–57. doi: 10.1021/acs.jpca.2c08023 (PMC9940216; doi:10.1021/acs.jpca.2c08023)
Supplement: Supplementary file 1 — jp2c08023_si_001.pdf [file jp2c08023_si_001.pdf]

Supporting Information

Electronic State Spectroscopy of Nitromethane and Nitroethane

Luiz V. S. Dalagnol [a], Márcio H. F. Bettega [a], Nykola C. Jones [b], Søren V. Hoffmann [b], Alessandra Souza Barbosa [a,*], and Paulo Limão-Vieira [a,c,*]

[a] Departamento de Física, Universidade Federal do Paraná, Caixa Postal 19044, 81531-980 Curitiba, Paraná, Brazil

[b] ISA, Department of Physics and Astronomy, Aarhus University, Ny Munkegade 120, DK-8000, Aarhus C, Denmark

[c] Atomic and Molecular Collisions Laboratory, CEFITEC, Department of Physics, NOVA School of Science and Technology, Universidade NOVA de Lisboa, 2829-516 Caparica, Portugal

Figure captions

Figure S1. Ground-state geometries obtained at the TD-DFT level with the B3LYP/aug-cc-pVDZ basis set for nitromethane *eclipsed* (left figure) and *staggered* (right figure) conformers. Bond lengths are in Å and bond angles in (°).

Figure S2. Ground-state geometries obtained at the TD-DFT level with the B3LYP/aug-cc-pVDZ basis set for nitroethane *eclipsed* (left figure) and *staggered* (right figure) conformers. Bond lengths are in Å and bond angles in (°).

Figure S3. Ionic electronic ground-state geometry obtained at the TD-DFT level with the B3LYP/aug-cc-pVDZ basis set for nitromethane. Bond lengths are in Å and bond angles in (°).

Figure S4. Ionic electronic ground-state geometry obtained at the TD-DFT level with the B3LYP/aug-cc-pVDZ basis set for nitroethane. Bond lengths are in Å and bond angles in (°).

Figure S5. Representation of a selection of the molecular orbitals (TD-DFT/CAM-B3LYP/aug-cc-pVDZ) of nitromethane (C_S).

Figure S6. Representation of a selection of the molecular orbitals (TD-DFT/CAM-B3LYP/aug-cc-pVDZ) of nitroethane (C_S).

Figure S7. PECs for the ground and low-lying excited singlet states of CH_3NO_2 plotted as a function of the $R_{C–N}$ coordinate and calculated at the TD-DFT/B3LYP/aug-cc-pVDZ level of theory in the C_s symmetry group. See text for details.

Table caption

Table S1. The calculated vertical excitation energies (TD-DFT/B3LYP/aug-cc-pVDZ) and oscillator strengths of nitromethane *staggered* and *eclipsed* conformers. Energies in eV. In bold the most relevant contributions to the experimental spectrum.

Table S2. The calculated vertical excitation energies (TD-DFT/B3LYP/aug-cc-pVDZ) and oscillator strengths of nitroethane *staggered* and *eclipsed* conformers. Energies in eV. In bold the most relevant contributions to the experimental spectrum.

Table S3. The calculated vibrational frequencies (B3LYP/aug-cc-pVDZ) (TD-DFT/B3LYP/aug-cc-pVDZ) of nitromethane and nitroethane neutral and ionic electronic ground-states.

Figure S1. Ground-state geometries obtained at the TD-DFT level with the B3LYP/aug-cc-pVDZ basis set for nitromethane *eclipsed* (left figure) and *staggered* (right figure) conformers. Bond lengths are in Å and bond angles in (°).

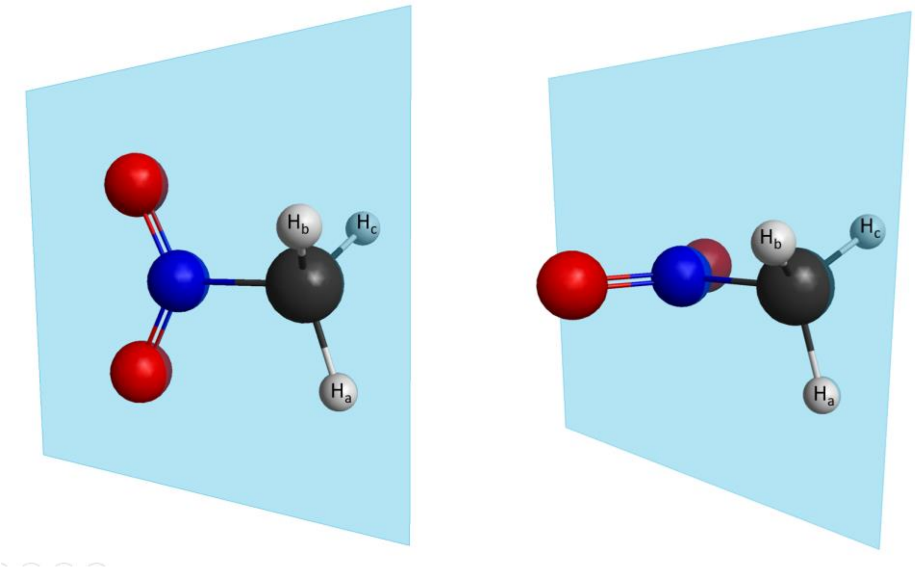

nitromethane	bond length (Å)	
	eclipsed	staggered
CN	1.500	1.500
NO	1.225	1.225
CH_a	1.091	1.096
$CH_{b,c}$	1.095	1.092
	angle (°)	
CNO	116.8	117.3
ONO	125.4	125.5
NCH_a	108.1	106.5
$NCH_{b,c}$	107.1	107.9

Electronic configuration of nitromethane $\tilde{X}\ ^1A'$ ground state:

Core orbitals: $(1a')^2$ $(2a')^2$ $(3a')^2$ $(4a')^2$

Valence orbitals: $(5a')^2$ $(6a')^2$ $(7a')^2$ $(8a')^2$ $(1a'')^2$ $(9a')^2$ $(10a')^2$ $(11a')^2$ $(2a'')^2$ $(3a'')^2$ $(12a')^2$ $(13a')^2$

Unoccupied orbitals of nitromethane:

(4a″) (14a′) (15a′) (5a″) (16a′) (17a′) (6a″) (18a′) (19a′) (20a′)

Figure S2. Ground-state geometries obtained at the TD-DFT level with the B3LYP/aug-cc-pVDZ basis set for nitroethane *eclipsed* (left figure) and *staggered* (right figure) conformers. Bond lengths are in Å and bond angles in (°).

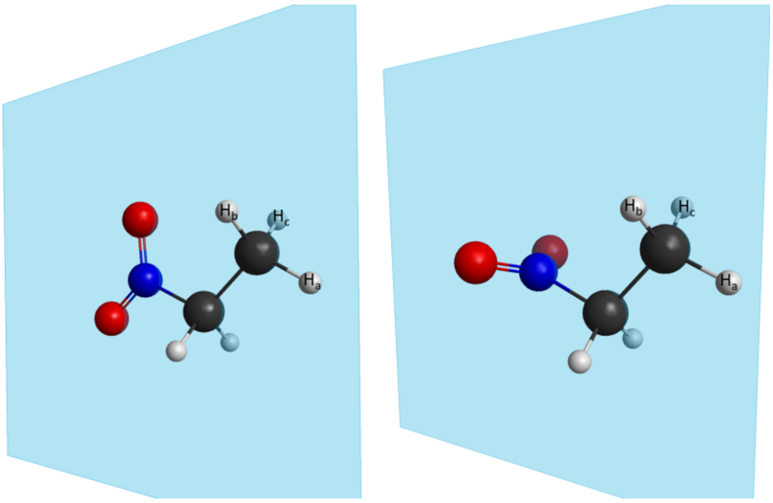

nitroethane	bond length (Å)	
	eclipsed	staggered
CN	1.520	1.510
CC	1.517	1.526
NO	1.225	1.226
C_1H	1.097	1.094
C_2H_a	1.099	1.098
$C_2H_{b,c}$	1.096	1.097
	angle (°)	
ONC	116.1	117.4
ONO	125.2	125.3
NCH	104.2	105.7
NCC	113.7	109.7
CCH_a	108.3	108.9
$CCH_{b,c}$	111.5	110.9

Electronic configuration of nitroethane $\tilde{X}$ $^1A'$ ground state:

Core orbitals: $(1a')^2$ $(2a')^2$ $(3a')^2$ $(4a')^2$ $(5a')^2$

Valence orbitals: $(6a')^2$ $(7a')^2$ $(8a')^2$ $(9a')^2$ $(10a')^2$ $(1a'')^2$ $(11a')^2$ $(12a')^2$ $(2a'')^2$ $(13a')^2$ $(14a')^2$ $(3a'')^2$ $(4a'')^2$ $(15a')^2$ $(16a')^2$

Unoccupied orbitals of nitroethane:

(5a″) (17a′) (18a′) (6a″) (19a′) (7a″) (8a″) (20a′) (21a′) (9a″)

Figure S3. Ionic electronic ground-state geometry obtained at the TD-DFT level with the B3LYP/aug-cc-pVDZ basis set for nitromethane. Bond lengths are in Å and bond angles in (°).

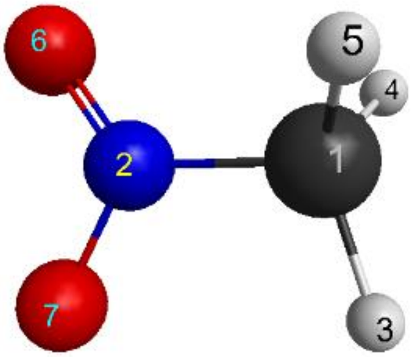

nitromethane cation	bond length (Å)
CN	1.475
NO(6)	1.198
NO(7)	1.278
CH(4/5)	1.102
CH(3)	1.099
	angle (°)
CNO(6)	127.2
CNO(7)	119.8
ONO	113.0
NCH(3)	107.4
NCH(4/5)	106.2
H(4)CH(5)	111.1

Figure S4. Ionic electronic ground-state geometry obtained at the TD-DFT level with the B3LYP/aug-cc-pVDZ basis set for nitroethane. Bond lengths are in Å and bond angles in (°).

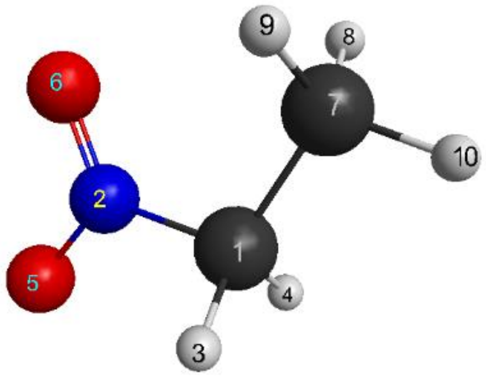

nitroethane cation	bond length (Å)
C(1)N	1.504
CC	1.522
NO(5)	1.282
NO(6)	1.196
C(1)H(3/4)	1.103
C(2)H(10)	1.100
C(2)H(9/8)	1.096
	angle (°)
O(5)NC	119.7
O(6)NC	126.7
ONO	113.6
NCH(3/4)	103.6
NCC	110.6
CCH(10)	105.5
CCH(8/9)	112.0

Figure S5. Representation of a selection of the molecular orbitals (TD-DFT/CAM-B3LYP/aug-cc-pVDZ) of nitromethane (C_S).

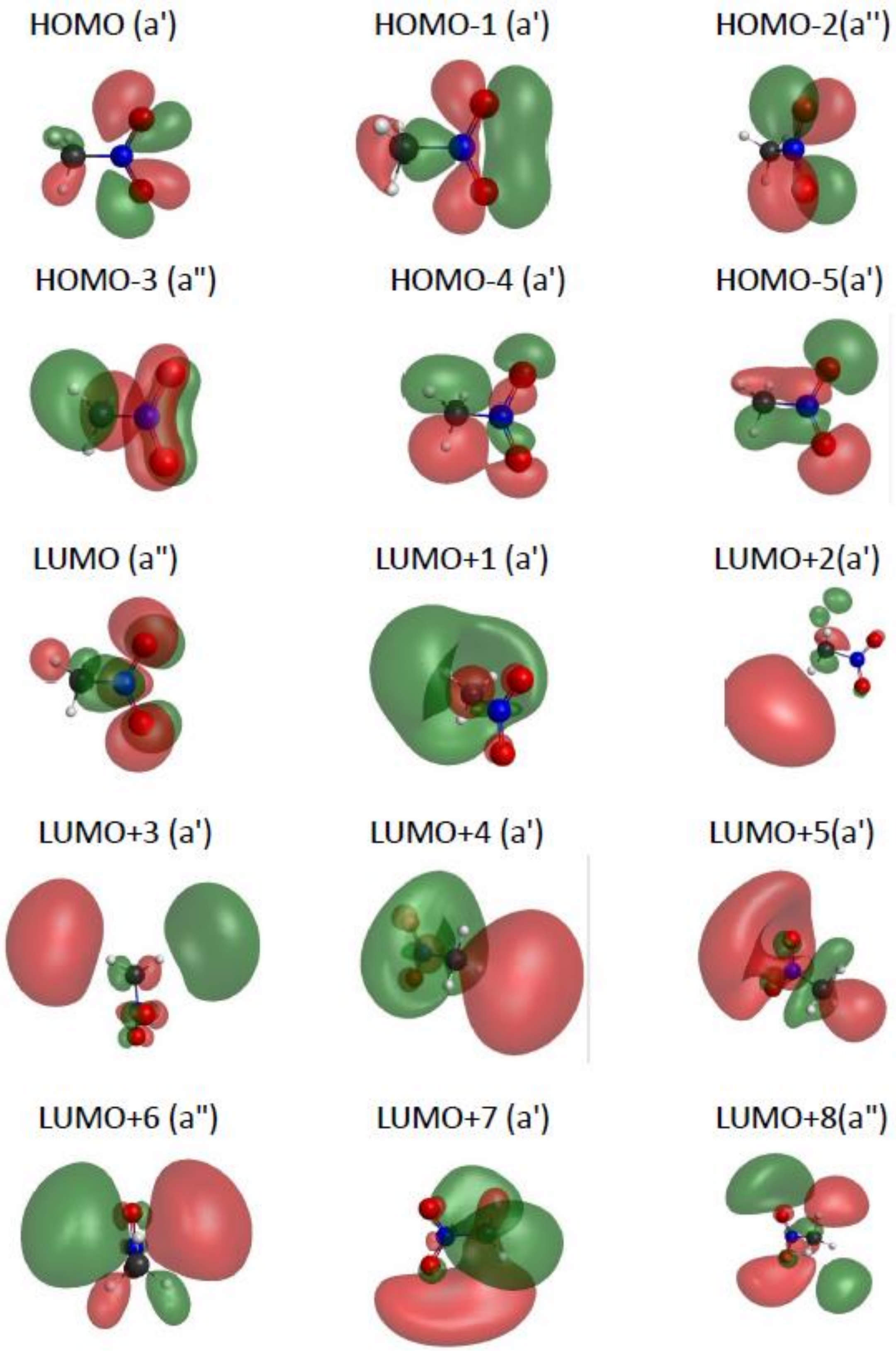

Figure S6. Representation of a selection of the molecular orbitals (TD-DFT/CAM-B3LYP/aug-cc-pVDZ) of nitroethane (C_S).

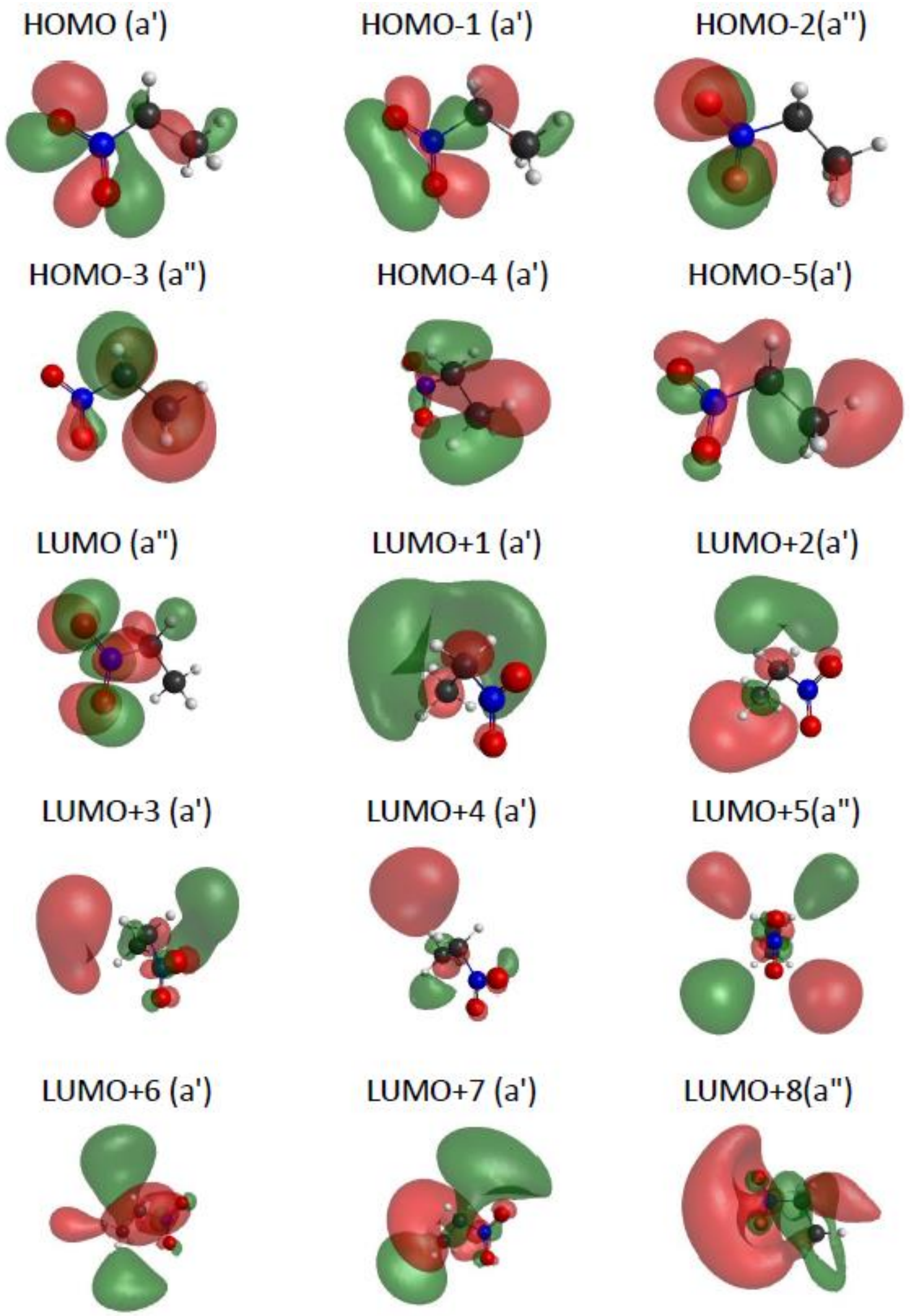

Figure S7. PECs for the ground and low-lying excited singlet states of CH_3NO_2 plotted as a function of the $R_{C–N}$ coordinate and calculated at the TD-DFT/B3LYP/aug-cc-pVDZ level of theory in the C_s symmetry group. See text for details.

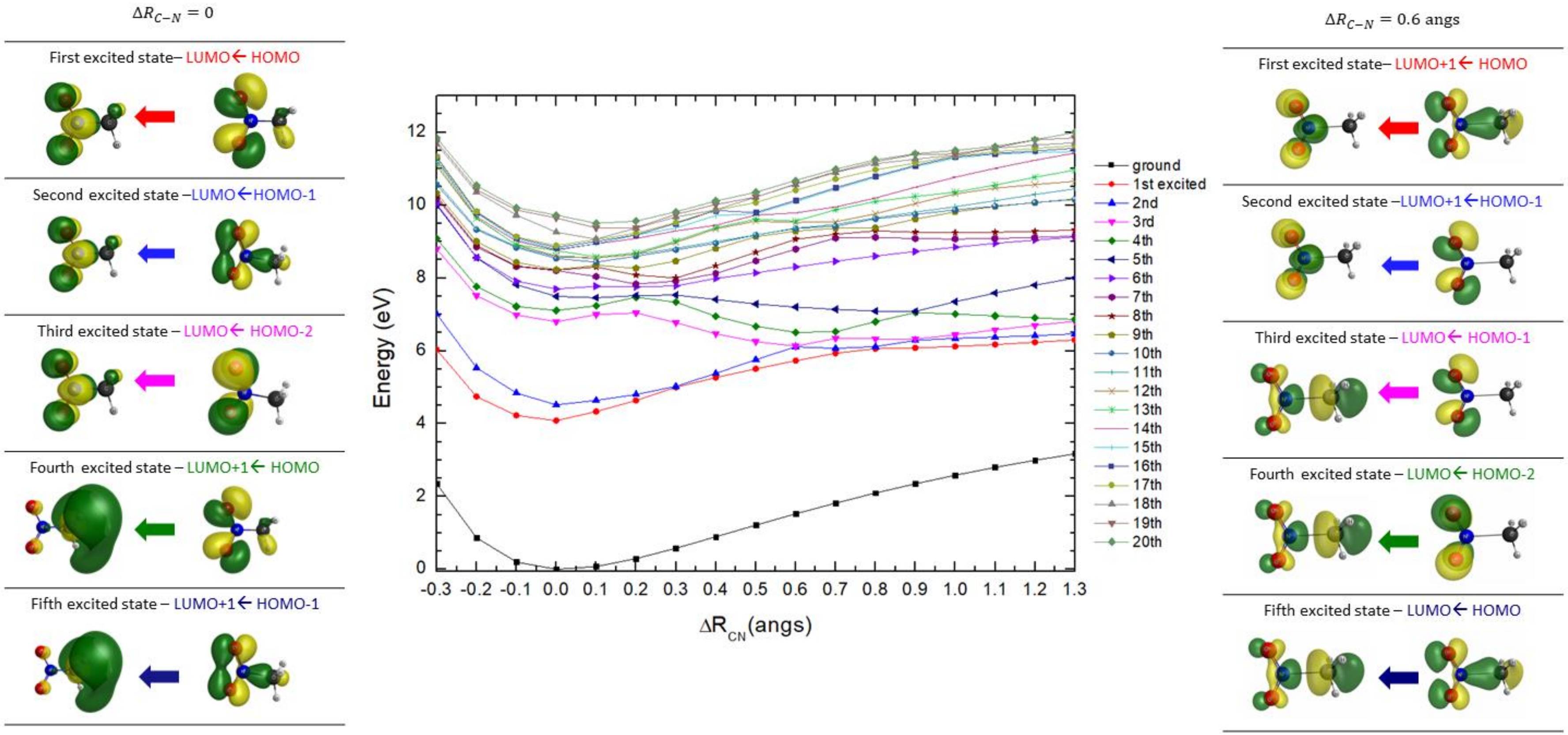

Table S1. The calculated vertical excitation energies (TD-DFT/B3LYP/aug-cc-pVDZ) and oscillator strengths of nitromethane *staggered* and *eclipsed* conformers. Energies in eV. In bold the most relevant contributions to the experimental spectrum.

staggered				*eclipsed*			
Energy	f_L	state	Major contributions	Energy	f_L	state	Major contributions
4.149	0.00004	A″	HOMO → LUMO (100%)	4.086	1.20E-05	A"	HOMO->LUMO (100%)
**4.605**	**0.00002**	**A″**	**H-1 → LUMO (100%)**	**4.516**	**2.90E-05**	**A'**	**H-1->LUMO (100%)**
**6.879**	**0.15459**	**A′**	**H-2 → LUMO (90%)**	**6.805**	**0.155973**	**A''**	**H-2->LUMO (92%)**
**7.100**	**0.00037**	**A′**	**HOMO → L+1 (95%)**	**7.110**	**2.00E-06**	**A''**	**HOMO->L+1 (96%)**
**7.510**	**0.03627**	**A′**	**H-1 → L+1 (97%)**	**7.494**	**0.036854**	**A'**	**H-1->L+1 (97%)**
7.714	0.00004	A"	H-2 → L+1 (98%)	7.703	1.27E-04	A"	H-2->L+1 (98%)
8.205	0.00017	A″	HOMO → L+3 (98%)	8.203	0.028972	A"	HOMO->L+2 (35%), HOMO->L+4 (60%)
8.205	0.01617	A′	HOMO → L+2 (74%), HOMO → L+4 (22%)	8.221	0.008147	A"	HOMO->L+2 (63%), HOMO->L+4 (34%)
**8.223**	**0.02625**	**A′**	**HOMO → L+2 (22%), HOMO → L+4 (72%)**	**8.228**	**0.005308**	**A'**	**HOMO->L+3 (96%)**
8.581	0.00655	A′	H-3 → LUMO (18%), H-1 → L+2 (24%), H-1 → L+4 (56%)	8.530	0.00491	A'	H-3->LUMO (29%), H-1->L+2 (20%), H-1->L+4 (49%)
8.594	0.00241	A″	H-1 → L+3 (98%)	8.586	0.003326	A'	H-1->L+2 (78%), H-1->L+4 (15%)
8.622	0.00833	A′	H-1 → L+2 (73%), H-1 → L+4 (18%)	8.607	0.010845	A"	H-1->L+3 (97%)
8.796	0.00116	A″	H-2 → L+2 (25%), H-2 → L+4 (72%)	8.760	0.002787	A"	H-4->LUMO (77%), H-2->L+4 (20%)
8.835	0.00013	A″	H-4 → LUMO (82%), H-2 → L+2 (15%)	8.771	5.77E-04	A"	H-4->LUMO (21%), H-2->L+2 (10%), H-2->L+4 (67%)
**8.851**	**0.10584**	**A′**	**H-3 → LUMO (70%), H-1 → L+4 (21%)**	**8.778**	**0.09112**	**A'**	**H-3->LUMO (55%), H-2->L+3 (10%), H-1->L+4 (29%)**
8.854	0.00726	A″	H-4 → LUMO (16%), H-2 → L+2 (59%), H-2 → L+4 (24%)	8.844	0.014642	A'	H-2->L+3 (89%)
8.909	0.00331	A′	H-2 → L+3 (91%)	8.891	0.001831	A"	H-2->L+2 (82%), H-2->L+4 (10%)
9.298	0.00771	A′	HOMO →L+5 (90%)	9.262	0.008218	A"	HOMO->L+5 (89%)
**9.702**	**0.20343**	**A′**	**H-1 → L+5 (79%), HOMO → L+8 (14%)**	**9.652**	**0.198201**	**A'**	**H-1->L+5 (88%)**
9.754	0.00017	A″	HOMO → L+6 (93%)	9.715	0.001943	A"	HOMO->L+6 (94%)
9.787	0.00164	A′	HOMO → L+7 (81%)	9.736	8.20E-05	A"	H-2->L+5 (98%)
9.798	0.00014	A″	H-2 → L+5 (98%)	9.817	0.004978	A'	HOMO->L+7 (83%)
9.942	0.00391	A′	H-1 → L+5 (10%), HOMO →L+8 (79%)	9.998	0.002392	A'	HOMO->L+8 (93%)
10.165	0.00000	A″	H-1 → L+6 (93%)	10.112	5.89E-04	A'	H-1->L+6 (89%)

10.194	0.01127	A′	H-1 → L+7 (85%)	10.205	0.021554	A"	H-1->L+7 (83%)
10.255	0.00263	A′	H-1 → L+8 (13%), HOMO → L+9 (83%)	10.243	0.001302	A"	H-1->L+8 (12%), HOMO->L+9 (73%)
10.330	0.01440	A″	H-2 → L+7 (71%), H-2 → L+8 (23%)	10.315	0.00489	A"	H-2->L+6 (89%)
10.353	0.04337	A′	H-1 → L+8 (75%), HOMO → L+9 (15%)	10.356	0.020728	A'	H-2->L+7 (84%), H-2->L+8 (10%)
10.453	0.00279	A′	H-2 → L+6 (92%)	10.380	0.031197	A"	H-1->L+8 (81%), HOMO->L+9 (16%)
10.516	0.00287	A″	H-2 → L+7 (19%), H-2 → L+8 (67%)	10.556	3.77E-04	A"	H-5->LUMO (91%)
10.571	0.00532	A″	HOMO → L+10 (87%)	10.561	0.066857	A'	H-3->L+1 (96%)
10.574	0.06421	A″	H-3 → L+1 (86%)	10.599	0.002281	A"	HOMO->L+10 (92%)
10.667	0.00044	A″	H-5 → LUMO (86%)	10.605	0.004208	A'	H-2->L+8 (59%), H-1->L+9 (33%)
**10.696**	**0.03473**	**A′**	**H-1 → L+9 (93%)**	**10.686**	**0.027099**	**A'**	**H-2->L+8 (27%), H-1->L+9 (61%)**
10.883	0.00035	A″	H-2 → L+9 (98%)	10.851	1.92E-04	A"	H-2->L+9 (98%)
10.931	0.00545	A′	HOMO → L+11 (85%)	10.889	0.003656	A'	H-6->LUMO (87%)
**10.960**	**0.02397**	**A″**	**H-1 → L+10 (90%)**	**10.954**	**0.021018**	**A'**	**H-1->L+10 (84%)**
11.022	0.00000	A″	H-6 → LUMO (87%)	10.976	0.012731	A"	HOMO->L+11 (65%), HOMO->L+13 (22%)
11.078	0.07801	A′	H-4 → L+1 (95%)	11.071	0.071166	A"	H-4->L+1 (91%)
11.151	0.00024	A″	HOMO → L+12 (85%)	11.083	0.001991	A'	HOMO->L+12 (84%)

Table S2. The calculated vertical excitation energies (TD-DFT/B3LYP/aug-cc-pVDZ) and oscillator strengths of nitroethane *staggered* and *eclipsed* conformers. Energies in eV. In bold the most relevant contributions to the experimental spectrum.

staggered				*eclipsed*			
Energy	f_L	state	Major contributions	Energy	f_L	state	Major contributions
4.200	0.00003	A″	HOMO → LUMO (99%)	4.201	2.50E-05	A"	HOMO->LUMO (99%)
**4.605**	**0.00006**	**A″**	**H-1 → LUMO (99%)**	**4.605**	**6.20E-05**	**A''**	**H-1->LUMO (99%)**
**6.789**	**0.11079**	**A′**	**H-2 → LUMO (86%)**	**6.789**	**0.110791**	**A'**	**H-2->LUMO (86%)**
7.033	0.01808	A′	HOMO → L+1 (95%)	7.034	0.018083	A'	HOMO->L+1 (95%)
7.338	0.00038	A′	H-3 → LUMO (22%), H-1 → L+1 (75%)	7.339	3.80E-04	A'	H-3->LUMO (22%), H-1->L+1 (75%)
**7.480**	**0.06883**	**A′**	**H-3 → LUMO (70%), H-1 → L+1 (20%)**	**7.481**	**0.068825**	**A'**	**H-3->LUMO (70%), H-1->L+1 (20%)**
7.599	0.00002	A″	H-2 → L+1 (96%)	7.600	2.20E-05	A"	H-2->L+1 (96%)
7.825	0.01007	A′	HOMO → L+2 (95%)	7.826	0.010074	A'	HOMO->L+2 (95%)
7.896	0.00231	A″	H-4 → LUMO (67%), HOMO → L+3 (30%)	7.897	0.00231	A"	H-4->LUMO (67%), HOMO->L+3 (30%)
7.913	0.00001	A″	H-4 → LUMO (30%), HOMO → L+3 (69%)	7.914	1.00E-05	A"	H-4->LUMO (30%), HOMO->L+3 (69%)
**8.087**	**0.02867**	**A′**	**H-1 → L+2 (20%), HOMO → L+4 (74%)**	**8.088**	**0.028666**	**A'**	**H-1->L+2 (20%), HOMO->L+4 (74%)**
8.171	0.00338	A′	H-1 → L+2 (77%), HOMO → L+4 (18%)	8.172	0.003378	A'	H-1->L+2 (77%), HOMO->L+4 (18%)
8.234	0.00076	A″	H-1 → L+3 (98%)	8.235	7.62E-04	A"	H-1->L+3 (98%)
8.387	0.00129	A″	H-2→ L+2 (97%)	8.388	0.001294	A"	H-2->L+2 (97%)
8.408	0.01056	A′	H-1 → L+4 (94%)	8.409	0.010562	A'	H-1->L+4 (94%)
8.484	0.00064	A″	H-5 → LUMO (95%)	8.485	6.37E-04	A"	H-5->LUMO (95%)
8.592	0.01010	A′	H-2 → L+3 (93%)	8.593	0.010104	A'	H-2->L+3 (93%)
8.644	0.00006	A″	H-2 →L+4 (94%)	8.645	5.60E-05	A"	H-2->L+4 (94%)
8.830	0.00049	A″	HOMO → L+5 (98%)	8.832	4.89E-04	A"	HOMO->L+5 (98%)
8.999	0.00881	A′	HOMO →L+6 (79%)	9.000	0.008806	A'	HOMO->L+6 (79%)
9.053	0.00164	A″	H-3 → L+1 (98%)	9.054	0.001635	A"	H-3->L+1 (98%)
**9.129**	**0.01501**	**A′**	**HOMO → L+7 (81%)**	**9.130**	**0.015012**	**A'**	**HOMO->L+7 (81%)**
9.155	0.00126	A″	H-1 → L+5 (98%)	9.156	0.001256	A"	H-1->L+5 (98%)

9.347	0.00440	A′	H-1 → L+6 (14%), HOMO → L+8 (60%)	9.348	0.004404	A'	H-1->L+6 (14%), HOMO->L+8 (60%)
9.384	0.02513	A′	H-2 → L+5 (13%), H-1 → L+6 (55%), HOMO → L+8 (19%)	9.385	0.02513	A'	H-2->L+5 (13%), H-1->L+6 (55%), HOMO->L+8 (19%)
9.414	0.00062	A′	H-6 → LUMO (10%), H-2 → L+5 (77%), H-1 → L+6 (11%)	9.415	6.22E-04	A'	H-6->LUMO (10%), H-2->L+5 (77%), H-1->L+6 (11%)
9.526	0.00080	A′	H-6 → LUMO (15%), H-1 → L+7 (76%)	9.527	7.96E-04	A'	H-6->LUMO (15%), H-1->L+7 (76%)
9.562	0.00307	A″	H-2 → L+6 (82%), H-2 → L+7 (11%)	9.563	0.003074	A"	H-2->L+6 (82%), H-2->L+7 (11%)
**9.664**	**0.05147**	**A′**	**H-6 → LUMO (29%), H-4 → L+1 (49%)**	**9.665**	**0.05147**	**A'**	**H-6->LUMO (29%), H-4->L+1 (49%)**
9.686	0.02981	A′	H-6 → LUMO (11%), H-1 → L+6 (10%), H-1 → L+8 (66%)	9.688	0.02981	A'	H-6->LUMO (11%), H-1->L+6 (10%), H-1->L+8 (66%)
9.727	0.00449	A″	H-2 → L+6 (10%), H-2 → L+7 (84%)	9.728	0.004494	A"	H-2->L+6 (10%), H-2->L+7 (84%)
**9.754**	**0.14702**	**A′**	**H-6 → LUMO (24%), H-4 → L+1 (48%), H-1 → L+8 (11%)**	**9.755**	**0.147022**	**A'**	**H-6->LUMO (24%), H-4->L+1 (48%), H-1->L+8 (11%)**
9.818	0.00028	A″	HOMO → L+9 (96%)	9.819	2.79E-04	A"	HOMO->L+9 (96%)
9.865	0.00054	A″	H-2 → L+8 (82%)	9.866	5.41E-04	A"	H-2->L+8 (82%)
9.878	0.06390	A″	H-3 → L+2 (95%)	9.879	0.063898	A"	H-3->L+2 (95%)
9.924	0.00200	A′	HOMO → L+10 (75%)	9.925	0.002004	A'	HOMO->L+10 (75%)
10.029	0.03196	A′	H-3 → L+3 (94%)	10.030	0.031956	A'	H-3->L+3 (94%)
10.140	0.00750	A″	H-3 → L+4 (39%), H-1 → L+9 (58%)	10.142	0.007499	A"	H-3->L+4 (39%), H-1->L+9 (58%)
10.159	0.00100	A″	H-3 → L+4 (59%), H-1 → L+9 (38%)	10.160	0.001004	A"	H-3->L+4 (59%), H-1->L+9 (38%)
10.212	0.02023	A′	HOMO → L+10 (10%), HOMO → L+11 (71%)	10.213	0.020234	A'	HOMO->L+10 (10%), HOMO->L+11 (71%)

Table S3. The calculated vibrational frequencies (B3LYP/aug-cc-pVDZ) (TD-DFT/B3LYP/aug-cc-pVDZ) of nitromethane and nitroethane neutral and ionic electronic ground-states.

	CH_3NO_2				$CH_3CH_2NO_2$			
	neutral		cation		neutral		cation	
	cm^{-1}	eV	cm^{-1}	eV	cm^{-1}	eV	cm^{-1}	eV
CH_3 twist	45.23	0.0056	45.05	0.0056	174.75	0.0217	163.78	0.0203
NO_2 rocking (in-p)	482.51	0.0598	461.45	0.0572	282.48	0.0350	203.77	0.0253
NO_2 rocking	630.05	0.0781	549.51	0.0681	586.94	0.0728	519.5	0.0644
NO_2 sym bend	675.40	0.0837	219.75	0.0272	632.08	0.0784	300.96	0.0373
CN str	958.82	0.1189	703.95	0.0873	878.14	0.1089	619.83	0.0769
CH_3 rock (in-p)	1114.63	0.1382	1026.44	0.1273	1110.81	0.1377	1147.59	0.1423
CH_3 rock	1140.43	0.1414	1102.72	0.1367	1138.37	0.1412	1124.53	0.1394
CH_3 sym bend	1403.69	0.1741	1363.27	0.1690	1389.67	0.1723	1380.62	0.1712
CH_3 a-sym bend (in-p)	1443.08	0.1789	1410.03	0.1748	1474.01	0.1828	1463.8	0.1815
CH_3 a-sym bend	1462.64	0.1814	1401.58	0.1738	1453.54	0.1802	1418.9	0.1759
NO_2 sym str	1493.67	0.1852	1561.90	0.1937	1425.23	0.1767	1555.32	0.1929
NO_2 a-sym str	1689.66	0.2095	1162.72	0.1442	1626.33	0.2017	996.45	0.1236
CH_3 sym str	3106.66	0.3852	3013.43	0.3737	3051.96	0.3784	3045.83	0.3777
CH_3 a-sym str	3205.97	0.3975	3117.42	0.3866	3150.35	0.3906	3158.97	0.3917
CH_3 a-sym str (in-p)	3238.86	0.4016	3147.87	0.3903	3125.93	0.3876	3127.21	0.3878
